# Effect of adherence to primaquine on the risk of *Plasmodium vivax* recurrence: a WorldWide Antimalarial Resistance Network systematic review and individual patient data meta-analysis

**DOI:** 10.1186/s12936-023-04725-w

**Published:** 2023-10-10

**Authors:** Parinaz Mehdipour, Megha Rajasekhar, Saber Dini, Sophie Zaloumis, Tesfay Abreha, Ishag Adam, Ghulam Rahim Awab, J. Kevin Baird, Larissa W. Brasil, Cindy S. Chu, Liwang Cui, André Daher, Margarete do Socorro M Gomes, Lilia Gonzalez‑Ceron, Jimee Hwang, Harin Karunajeewa, Marcus V. G. Lacerda, Simone Ladeia-Andrade, Toby Leslie, Benedikt Ley, Kartini Lidia, Alejandro Llanos-Cuentas, Rhea J. Longley, Wuelton Marcelo Monteiro, Dhelio B. Pereira, Komal Raj Rijal, Kavitha Saravu, Inge Sutanto, Walter R. J. Taylor, Pham Vinh Thanh, Kamala Thriemer, José Luiz F. Vieira, Nicholas J. White, Lina M. Zuluaga-Idarraga, Philippe J. Guerin, Ric N. Price, Julie A. Simpson, Robert J. Commons, the WWARN Vivax Adherence Study Group, Bipin Adhikari, Mohammad Shafiul Alam, Ashenafi Assefa, Sarah C. Boyd, Nguyen Hoang Chau, Nicholas P. J. Day, Tamiru Shibiru Degaga, Arjen M. Dondorp, Annette Erhart, Marcelo U. Ferreira, Prakash Ghimire, Justin A. Green, Wasif Ali Khan, Gavin C. K. W. Koh, Asrat Hailu Mekuria, Ivo Mueller, Mohammad Nader Naadim, Erni J. Nelwan, Francois Nosten, Ayodhia Pitaloka Pasaribu, Sasithon Pukrittayakamee, Mark Rowland, Jetsumon Sattabongkot, Kasia Stepniewska, Guilherme Suarez‑Kurtz, Lorenz von Seidlein, Charles J. Woodrow, Adugna Woyessa

**Affiliations:** 1 Centre for Epidemiology and Biostatistics, Melbourne School of Population and Global Health, The University of Melbourne, Melbourne, Australia; 2 ICAP, Columbia University Mailman School of Public Health, Addis Ababa, Ethiopia; 3 Department of Obstetrics and Gynecology, Unaizah College of Medicine and Medical Sciences, Qassim University, Unaizah, Saudi Arabia; 4 Mahidol Oxford Tropical Medicine Research Unit (MORU), Faculty of Tropical Medicine, Mahidol University, Bangkok, Thailand; 5 Nangarhar Medical Faculty, Nangarhar University, Jalalabad, Afghanistan; 6 Oxford University Clinical Research Unit Indonesia, Faculty of Medicine, Universitas Indonesia, Jakarta, Indonesia; 7 Centre for Tropical Medicine and Global Health, Nuffield Department of Medicine, Oxford University, Oxford, UK; 8 Diretoria de Ensino E Pesquisa, Fundação de Medicina Tropical Dr. Heitor Vieira Dourado, Manaus, AM Brazil; 9 Programa de Pós‑Graduação em Medicina Tropical, Universidade Do Estado Do Amazonas, Manaus, AM Brazil; 10 Shoklo Malaria Research Unit, Mahidol Oxford Tropical Medicine Research Unit, Faculty of Tropical Medicine, Mahidol University, Mae Sot, Thailand; 11 Department of Internal Medicine, Morsani College of Medicine, University of South Florida, Tampa, FL USA; 12 Fiocruz Clinical Research Platform, Vice-Presidency of Research and Biological Collections, Oswaldo Cruz Foundation (FIOCRUZ), Rio de Janeiro, Brazil; 13 Superintendência de Vigilância Em Saúde Do Estado Do Amapá - SVS/AP, Macapá, Amapá Brazil; 14 Federal University of aMAPA, Universidade Federal Do Amapá - UNIFAP), Macapá, Amapá Brazil; 15 Regional Centre for Public Health Research, National Institute for Public Health, Tapachula, Chiapas, Mexico; 16 U.S. President’s Malaria Initiative, Malaria Branch, U.S. Centers for Disease Control and Prevention, Atlanta, GA USA; 17 Global Health Group, University of California San Francisco, San Francisco, USA; 18 Department of Medicine-Western Health, Melbourne Medical School, The University of Melbourne, St. Albans, VIC Australia; 19 Fundação de Medicina Tropical Dr Heitor Vieira Dourado, Manaus, Brazil; 20 Instituto Leônidas & Maria Deane, Fiocruz, Manaus, Brazil; 21 University of Texas Medical Branch, Galveston, USA; 22 Laboratory of Parasitic Diseases, Oswaldo Cruz Institute, Fiocruz, Rio de Janeiro, Brazil; 23 Global Health and Tropical Medicine, Institute of Hygiene and Tropical Medicine, Nova University of Lisbon, Lisbon, Portugal; 24 Department of Infectious and Tropical Diseases, London School of Hygiene and Tropical Medicine, London, UK; 25 HealthNet-TPO, Kabul, Afghanistan; 26 Global Health Division, Menzies School of Health Research and Charles Darwin University, Darwin, NT Australia; 27 Department of Pharmacology and Therapy, Faculty of Medicine and Veterinary Medicine, Universitas Nusa Cendana, Kupang, Indonesia; 28 Unit of Leishmaniasis and Malaria, Instituto de Medicina Tropical “Alexander Von Humboldt”, Universidad Peruana Cayetano Heredia, Lima, Peru; 29 Mahidol Vivax Research Unit, Faculty of Tropical Medicine, Mahidol University, Bangkok, Thailand; 30 Population Health and Immunity Division, Walter and Eliza Hall Institute of Medical Research, Melbourne, Australia; 31 Department of Medical Biology, University of Melbourne, Melbourne, Australia; 32 Universidade Do Estado Do Amazonas, Manaus, Brazil; 33 Centro de Pesquisa Em Medicina Tropical de Rondonia (CEPEM), Porto Velho, Brazil; 34 Fundação Universidade Federal de Rondonia (UNIR), Porto Velho, Brazil; 35 Central Department of Microbiology, Tribhuvan University, Kirtipur, Kathmandu, Nepal; 36 Department of Clinical Tropical Medicine, Faculty of Tropical Medicine, Mahidol University, Bangkok, Thailand; 37 Department of Infectious Diseases, Kasturba Medical College Manipal, Manipal Academy of Higher Education, Madhava Nagar, Manipal, Karnataka India; 38 Manipal Centre for Infectious Diseases, Prasanna School of Public Health, Manipal Academy of Higher Education, Madhava Nagar, Manipal, Karnataka India; 39 Department of Parasitology, Faculty of Medicine, University of Indonesia, Jakarta, Indonesia; 40 National Institute of Malariology, Parasitology and Entomology, Hanoi, Vietnam; 41 Federal University of Pará, Universidade Federal Do Pará - UFPA), Belém, Pará, Brazil; 42 Grupo Malaria, Facultad de Medicina, Universidad de Antioquia, Medellín, Colombia; 43 Facultad Nacional de Salud Publica, Universidad de Antioquia, Medellín, Colombia; 44 WorldWide Antimalarial Resistance Network (WWARN), Oxford, UK; 45 Infectious Diseases Data Observatory (IDDO), Oxford, UK; 46 WorldWide Antimalarial Resistance Network (WWARN), Asia-Pacific Regional Centre, Darwin, NT Australia; 47 General and Subspecialty Medicine, Grampians Health – Ballarat, Ballarat, Australia

**Keywords:** Malaria, *Plasmodium vivax*, Adherence, Primaquine, Rate of recurrence, Supervision

## Abstract

**Background:**

Imperfect adherence is a major barrier to effective primaquine radical cure of *Plasmodium vivax*. This study investigated the effect of reduced adherence on the risk of *P. vivax* recurrence.

**Methods:**

Efficacy studies of patients with uncomplicated *P. vivax* malaria, including a treatment arm with daily primaquine, published between January 1999 and March 2020 were identified. Individual patient data from eligible studies were pooled using standardized methodology. Adherence to primaquine was inferred from i) the percentage of supervised doses and ii) the total mg/kg dose received compared to the target total mg/kg dose per protocol. The effect of adherence to primaquine on the incidence of *P. vivax* recurrence between days 7 and 90 was investigated by Cox regression analysis.

**Results:**

Of 82 eligible studies, 32 were available including 6917 patients from 18 countries. For adherence assessed by percentage of supervised primaquine, 2790 patients (40.3%) had poor adherence (≤ 50%) and 4127 (59.7%) had complete adherence. The risk of recurrence by day 90 was 14.0% [95% confidence interval: 12.1–16.1] in patients with poor adherence compared to 5.8% [5.0–6.7] following full adherence; p = 0.014. After controlling for age, sex, baseline parasitaemia, and total primaquine dose per protocol, the rate of the first recurrence was higher following poor adherence compared to patients with full adherence (adjusted hazard ratio (AHR) = 2.3 [1.8–2.9]). When adherence was quantified by total mg/kg dose received among 3706 patients, 347 (9.4%) had poor adherence, 88 (2.4%) had moderate adherence, and 3271 (88.2%) had complete adherence to treatment. The risks of recurrence by day 90 were 8.2% [4.3–15.2] in patients with poor adherence and 4.9% [4.1–5.8] in patients with full adherence; p < 0.001.

**Conclusion:**

Reduced adherence, including less supervision, increases the risk of vivax recurrence.

**Supplementary Information:**

The online version contains supplementary material available at 10.1186/s12936-023-04725-w.

## Background

Early diagnosis and effective treatment of malaria are critical to achieving the ambitious goals of reducing malaria case incidence and mortality rates by at least 90% and eliminating malaria in at least 35 countries by 2030 [1, 2]. Anti-malarial efficacy studies usually focus on the safety and efficacy of anti-malarial drugs in a supervised setting; whereas, adherence and effectiveness of anti-malarial regimens are rarely considered [3].

Forty percent of the world’s population is at risk of *Plasmodium vivax* infection, with 5–15 million clinical episodes of vivax malaria each year [2, 4]. Treating infections with vivax malaria is challenging because *P. vivax* forms dormant liver stages that can reactivate to cause bloodstream infections (relapses) weeks to months after the initial infection. Treating both the blood and liver stages of vivax malaria has major public health benefits including reducing the morbidity and mortality associated with vivax malaria [5].

Primaquine, the most widely used anti-malarial that kills the liver stage of *P. vivax*, is administered over 7 to 14 days. Since primaquine treatment is completed well after the clinical symptoms of malaria have resolved, adherence to a complete course of treatment is often poor [6, 7]. When treatment is unsupervised the risk of *P. vivax* recurrence increases [5, 7, 8].

To better understand the impact of reduced adherence and inform strategies for improving the effectiveness of primaquine radical cure regimens, an individual patient data pooled meta-analysis of prospective *P. vivax* clinical efficacy studies was undertaken to investigate the effect of reduced adherence on the risk of *P. vivax* recurrence between days 7 and 90 and the key patient factors that contribute to reduced adherence.

## Methods

### Search strategy and selection criteria

A systematic review of all prospective *P. vivax* clinical efficacy studies was updated as previously described [9, 10]. In brief, MEDLINE, Web of Science, Embase, and Cochrane Central were searched according to the Preferred Reporting Items for Systematic Reviews and Meta-Analyses statement [11] (Additional file 1: checklist S1) from January 1, 1999, to March 3, 2020, in any language. Prospective therapeutic efficacy studies of uncomplicated *P. vivax* malaria that were included comprised randomized and non-randomized trials and prospective cohort studies with a minimum of 28 days of active follow-up that administered daily primaquine within three days of commencing chloroquine or an artemisinin-based combination therapy as a blood schizontocidal treatment. Studies were excluded if adjunctive drugs were given or if primaquine had been administered intermittently (Additional file 1: Box S1).

Investigators of eligible studies were contacted to include their data, in addition to requesting data from unpublished or ongoing clinical studies if available. Individual patient data were uploaded into the WorldWide Antimalarial Resistance Network (WWARN) repository, curated, and standardized using the WWARN Data Management and Statistical Analysis Plans [12].

### Procedures

Individual patient records were excluded if primaquine dosing regimens were intermittent (i.e., weekly, or monthly treatment) or data on supervision and dosing were unavailable. Patients with no data on age, sex, body weight, and baseline parasitaemia were also excluded. Primaquine mg/kg doses were calculated from the number of tablets, or the mg doses given to each patient. If the daily tablet or mg dose was not available, doses were derived from the expected mg daily dose according to the weight-based or age-based dosing regimen in the study protocol. Vomited doses were considered to be replaced if redosing data were available and doses were re-administered on the same day.

The exposure of interest, adherence to primaquine, was calculated based on supervision status and dosing information (Table 1). For the main analysis, the metric for adherence was derived from the level of supervision of primaquine intake for each patient. In a subset of patients with more detailed information available, two additional metrics for adherence were derived: actual doses of primaquine administered, and the total mg/kg dose administered. The three metrics were calculated for each patient based on a comparison between actual doses recorded as being administered per individual and the target dose according to the study protocol. The primary metric of adherence was the level of supervision which was defined as the number of directly observed (supervised) doses divided by the total number of administered or expected doses per protocol. Data on supervision were derived from doses recorded as being administered where possible, or if not, were assumed from expected doses as per the study protocol. The secondary measures of adherence were calculated based on dosing information for each patient. In studies in which the daily administration of all drug doses was recorded, the percentage of doses received by each patient was calculated from the number of doses administered divided by the number of doses expected per protocol. The second assessment of adherence by dosing information was derived using the total mg/kg dose administered as a proxy for adherence in studies in which daily dose data were not recorded. Adherence was calculated from the total mg/kg dose administered divided by the total target mg/kg dose per protocol (i.e., maximum was 100%).

**Table 1:** Adherence to primaquine definitions in the study

Adherence by	Metric	Proportion (%)	Categories (as per distribution)
Supervision status	Supervision	$\frac{\mathrm{number\, of \,daily\, doses\, supervised}}{\mathrm{ total\, number\, of\, daily\, doses\, administered\, or\, expected}}$	0- ≤ 50% > 50- < 90% ≥ 90%
Dosing information	Actual doses of primaquine administered	$\frac{\mathrm{number\, of\, daily\, doses\, administered }}{\mathrm{ total\, number\, of\, daily\, doses\, expected}}$	< 90% ≥ 90%
	Total mg/kg dose administered	$$\frac{{{\text{total }}{\raise0.7ex\hbox{${{\text{mg}}}$} \!\mathord{\left/ {\vphantom {{{\text{mg}}} {{\text{kg}}}}}\right.\kern-0pt} \!\lower0.7ex\hbox{${{\text{kg}}}$}}{\text{ dose administered}}}}{{{\text{total }}{\raise0.7ex\hbox{${{\text{mg}}}$} \!\mathord{\left/ {\vphantom {{{\text{mg}}} {{\text{kg}}}}}\right.\kern-0pt} \!\lower0.7ex\hbox{${{\text{kg}}}$}}{\text{ dose expected}}}}$$	0- ≤ 50% > 50- < 90% ≥ 90%

Based on their distributions, adherence by supervision or percentage of total mg/kg dose received were categorized into three groups: 0 to ≤ 50% (poor adherence), > 50% to < 90% (moderate adherence), and ≥ 90% (full adherence). As most patients had full adherence using information of actual doses of primaquine administered, this measure was categorized into two groups: imperfect adherence (< 90%) and full adherence (≥ 90%). There were very few patients in both ≤ 50% and > 50- < 90% groups for adherence by actual dosing information.

Transmission intensity at each study site was categorized as low (≤ 1 case per 1000 person-years), moderate (> 1 and < 10 cases per 1000 person-years), or high (≥ 10 cases per 1000 person-years) based on transmission estimates obtained from the Malaria Atlas Project [13, 14]. Study sites were also categorized as having long or short *P. vivax* relapse periodicity according to their geographical location [14], with regions with short relapse periodicity defined as having a median time to patent relapse of ≤ 47 days [14]. The elimination half-life of the schizontocidal anti-malarial drug used was categorized as rapid (< 1 day), intermediate (1–7 days), or slow (> 7 days), based on the longest acting partner drug in combination therapies [15]. The total dose of primaquine was categorized as very low dose (< 2.5 mg/kg), low dose (2.5– < 5 mg/kg), and high dose (≥ 5 mg/kg) [16]. In studies where haematocrit was available but not haemoglobin, haematocrit was converted to haemoglobin using the formula: Haemoglobin = (Haematocrit – 5.62)/2.60 [17].

### Outcomes

The primary outcome was *P. vivax* recurrence between day 7 and day 90. The secondary outcome was *P. vivax* recurrence between day 7 and day 42.

### Statistical analysis

Summaries of baseline patient characteristics were provided for different measures of adherence to primaquine. The risk of the first *P. vivax* recurrence between days 7 and 90 (and days 7 and 42) was calculated for the primary and secondary measures of adherence (based on supervision and the total mg/kg dose of primaquine administered) using Kaplan–Meier survival analysis. Patients were right-censored at the day they were last seen, the day prior to a more than 60-day gap between blood smears, the last day of study follow-up or the day of outcome (first recurrence), depending on whichever came first [12].

Cox’s proportional hazards regression analysis was used to estimate the association between adherence category (based on supervision status or total mg/kg dose administered) and the rate of *P. vivax* recurrence during follow-up. Adherence by actual dose of primaquine administered was not investigated since over 97% of individuals had full adherence (≥ 90%). The Cox model with the adherence metric being the level of supervision as the exposure of interest was adjusted for age, sex, baseline parasitaemia and target total mg/kg dose per protocol. A random intercept for the study site was not included due to collinearity with supervision status. The Cox model with the adherence metric being the total mg/kg dose administered as the exposure of interest included age, sex, and baseline parasitaemia, with a random intercept for the study site to account for the unobserved heterogeneity in patients’ hazard related to different sites. The expected mg/kg total dose per protocol was not included due to correlation with the exposure (total mg/kg dose administered). Body weight of the patients was not included as a confounder in the multivariable analysis due to collinearity with age and indirect inclusion through total mg/kg dose. The elimination half-life of schizontocidal treatment was not included in the regression analysis as the majority of patients (96%) were prescribed a slowly eliminated anti-malarial drug (half-life > 7 days). The proportional hazards assumption was tested visually and using Schoenfeld residuals. If non-proportional hazards were present for the adherence exposure of interest, interactions between adherence and time were assessed.

A separate analysis was undertaken to identify key factors contributing to imperfect patient adherence in studies in which the actual dose of primaquine administered was recorded. Adherence by supervision was not considered in this analysis due to the collinearity of supervision with study sites. Adherence by total mg/kg dose was not included in this analysis as this measurement is strongly collinear with age and weight. Imperfect adherence was defined as adherence < 90% by the actual dose administered information. The univariable association between imperfect adherence and age as a categorical variable, sex, the daily dose of primaquine, start day of primaquine, expected primaquine duration, vomiting, baseline parasitaemia density, and baseline fever as a marker of clinical illness severity were analysed by logistic regression, with study site included as a random effect.

To assess the risk of bias, baseline characteristics of included studies were compared with targeted studies that were not included. Heterogeneity of studies was assessed by removal of one study site at a time and calculation of the coefficient of variation around parameter estimates. A post hoc sensitivity analysis was undertaken with level of supervision as a variable of interest, repeating the Cox model in patient data i) collected in randomized studies and ii) in non-randomized studies.

Statistical analyses were done in Stata (version 16.0) and R (version 4.1.3), according to a statistical analysis plan [18]. The review is registered in PROSPERO, number CRD42020173816.

## Results

**Fig. 1:**
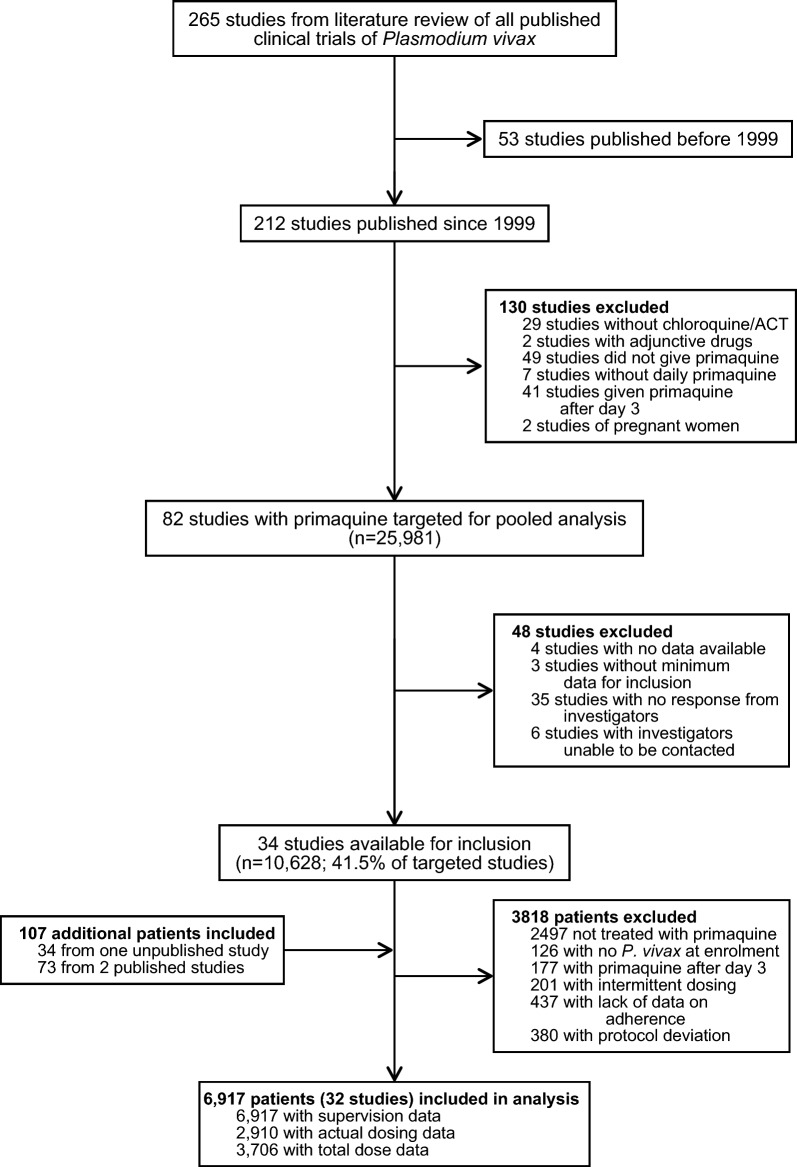
Study profile

Of 265 published *P. vivax* efficacy studies since 1960, 82 eligible studies were published between January 1, 1999, and March 3, 2020, and included patients treated with primaquine (Fig. 1; Additional file 1: Tables S1–S3). Individual patient data were available from 34 (41.5%) of the targeted published studies including 10,628 patients. Additional patient data were available for 107 patients from one unpublished and two published studies (Additional file 1: Table S1). Of these 10,735 patients, 3818 (35.6%) patients were excluded because of protocol deviations and not being treated with primaquine, leaving 6917 patients from 32 studies and 18 countries (Fig. 1 and Additional file 1: Figs. S1–S3) [19–49]. Of the 6917 patients included in the analysis, all had data on supervision, 2910 (42.1%) had information on actual daily doses administered, and 3706 (53.6%) had data on the total mg/kg dose administered. The baseline characteristics of patients of studies targeted for inclusion, but not included in the pooled analysis, were similar to patients in the included studies although they were enrolled less recently and were older (Additional file 1: Table S4).

### Adherence by supervision

A total of 6917 (100%) patients were included in the analysis for primaquine adherence defined according to supervision, of whom 4127 (59.7%) patients had ≥ 90% adherence, 2790 (40.3%) had ≤ 50% adherence and no patients had adherence between > 50 and < 90%. The median age of patients was 19.0 years (interquartile range (IQR) [11.0–32.0]; range 9 months to 94 years), with 452 (6.5%) aged younger than 5 years (Table 2). In total 4904 (70.9%) patients were from the Asia–Pacific region, compared with 1338 (19.4%) from the Americas and 672 (9.7%) from Africa. Patients with ≤ 50% adherence to primaquine defined according to supervision were older, more likely to come from regions with low relapse periodicity, to start primaquine after day 1 and have a lower baseline parasitaemia (Table 2).

**Table 2:** Demographic and baseline characteristics for adherence by supervision

	Adherence by supervision (%)		Overall (N = 6917)
	≤ 50 (N = 2790)	≥ 90 (N = 4127)	
Sex			
Female	1036 (37.1%)	1493 (36.2%)	2529 (36.6%)
Male	1754 (62.9%)	2630 (63.8%)	4384 (63.4%)
Age (years)			
Median [IQR]	23.0 (11.0–37.9)	17.1 (11.0–29.0)	19.0 (11.0–32.0)
< 5	191 (6.8%)	261 (6.3%)	452 (6.5%)
5–15	726 (26.0%)	1409 (34.2%)	2135 (30.9%)
> = 15	1872 (67.1%)	2453 (59.5%)	4325 (62.6%)
Relapse periodicity			
Low periodicity	1580 (56.6%)	1489 (36.1%)	3069 (44.4%)
High periodicity	1210 (43.4%)	2635 (63.9%)	3845 (55.6%)
Geographical region			
Africa	207 (7.4%)	465 (11.3%)	672 (9.7%)
Americas	722 (25.9%)	616 (14.9%)	1338 (19.4%)
Asia–Pacific	1861 (66.7%)	3043 (73.8%)	4904 (70.9%)
Transmission intensity of study site			
Low	261 (9.4%)	1526 (37.0%)	1787 (25.8%)
Moderate	1239 (44.4%)	1427 (34.6%)	2666 (38.6%)
High	1290 (46.2%)	1171 (28.4%)	2461 (35.6%)
Blood-stage drug elimination half-life			
Rapid, < 1 day	50 (1.8%)	0 (0.0%)	50 (0.7%)
Intermediate, 1–7 days	217 (7.8%)	31 (0.8%)	248 (3.6%)
Slow, > 7 days	2523 (90.4%)	4096 (99.2%)	6619 (95.7%)
Primaquine regimen duration			
< 14 days	1136 (40.7%)	1967 (47.7%)	3103 (44.9%)
14 days	1654 (59.3%)	2160 (52.3%)	3814 (55.1%)
Start day of primaquine			
Day 0/1	2025 (72.6%)	3990 (96.7%)	6015 (87.0%)
Day 2/3	765 (27.4%)	137 (3.3%)	902 (13.0%)
Planned primaquine total dose			
Very low dose	78 (2.8%)	208 (5.0%)	286 (4.1%)
Low dose	2641 (94.7%)	1708 (41.4%)	4349 (62.9%)
High dose	71 (2.5%)	2206 (53.5%)	2277 (32.9%)
Malnutrition			
Yes	19 (18.1%)	64 (24.6%)	83 (22.7%)
No	86 (81.9%)	196 (75.4%)	282 (77.3%)
Fever at baseline, temperature > 37.5 °C			
Yes	1222 (57.9%)	1615 (45.0%)	2837 (49.8%)
No	889 (42.1%)	1975 (55.0%)	2864 (50.2%)
Weight (kg)	57.0 (45.0–68.0)	47.0 (27.0–57.6)	50.0 (30.8–61.2)
Haemoglobin (g/dL)	12.7 (11.2–14.0)	12.7 (11.5–14.0)	12.7 (11.4–14.0)
Parasitaemia, parasites per μL	2520.0 (960.0–5802.0)	3189.8 (870.4–8923.0)	2938.5 (900.0–7500.0)

Data are presented as median (IQR) for continuous measures, and n (%) for categorical measures

IQR – Interquartile range; there were no patients with adherence by supervision between > 50- < 90%

Data were missing for the following variables: sex (4 patients in the ≥ 90% group), age (4 patients in ≥ 90% group and 1 patient in the ≤ 50% group), relapse periodicity, geographical region, and transmission intensity of study site (3 patients in the ≥ 90% group), planned total dose of primaquine (5 patients in the ≥ 90% group), malnutrition status – calculated for children aged < 5 years of age (86 patients in ≤ 50% group and 1 patient in the ≥ 90% group), fever (679 patients in ≤ 50% and 537 patients in the ≥ 90% group), baseline parasitaemia (122 patients in ≥ 90% group and 449 patient in the ≤ 50% group), baseline haemoglobin (518 patients in ≥ 90% group and 828 patient in the ≤ 50% group), weight (65 patients in ≥ 90% group and 807 patient in the ≤ 50% group)

**Fig. 2:**
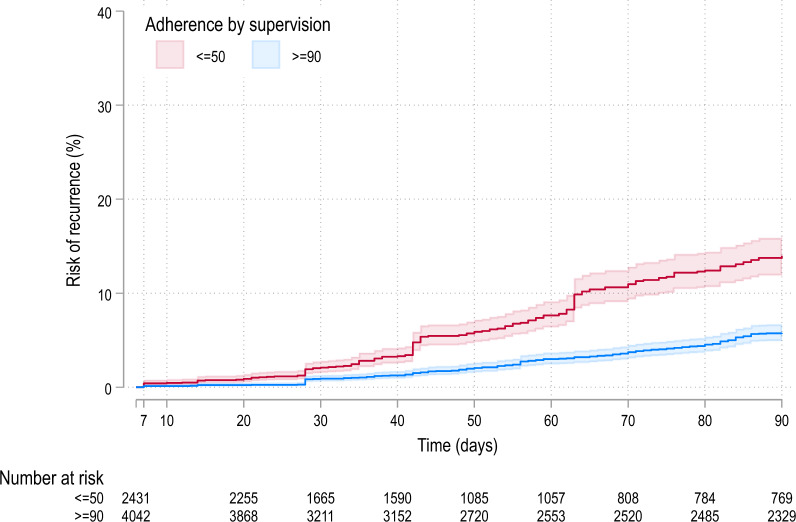
Risk of the first recurrence between days 7 and 90 in patients with adherence by supervision. The shaded areas represent 95% CIs. There were no patients with adherence by supervision between > 50– <90%.

Overall, 353 (5.1%) patients had *P. vivax* recurrence between days 7 and 90, with 186 recurrences in patients with ≤ 50% adherence and 167 recurrences in patients with ≥ 90% adherence. The cumulative risks of recurrence at day 42 were 4.7% [95% confidence interval (CI) 3.9–5.9] in patients with ≤ 50% adherence and 1.5% [1.2–1.9] in patients with ≥ 90% adherence; p = 0.014 (Fig. 2). The corresponding risks at 90 days were 14.0% [12.1–16.1] and 5.8% [5.0–6.7]; p < 0.001 (Fig. 2). Patients within a study site were either all poor adherers (< 50%) or all complete adherers (≥ 90%) (Additional file 1: Table S5 panel A).

After controlling for age, sex, baseline parasitaemia, and target primaquine total dose, the rate of the first recurrence between days 7 to 90 was higher following poor versus full adherence (adjusted hazard ratio (AHR) = 2.3, 95% CI [1.8–2.9]; p < 0.001 (Additional file 1: Table S6, Fig. S4). Results were similar in sensitivity analyses restricted to randomized controlled trials and observational studies (Additional file 1: Table S7). Sensitivity analyses in which one study site was removed at a time revealed no apparent bias relating to individual study sites from included studies (Additional file 1: Table S8).

### Adherence by total mg/kg dose administered

There were 3706 (53.6%) patients with data available on the total mg/kg dose administered, of whom 3271 (88.3%) patients had ≥ 90% adherence, 88 (2.4%) had > 50- < 90% adherence and 347 (9.4%) had ≤ 50% adherence. The median age was 17.4 years (IQR: 11.0–29.0; range from 9 months to 94 years), with 257 (6.9%) aged younger than 5 years (Additional file 1: Table S9). There were 2760 (74.5%) patients from the Asia–Pacific region, and an equal proportion of patients in the Americas (13.0%) and Africa (12.5%). Compared with patients with full adherence (≥ 90%), patients with poor adherence (≤ 50%) defined by the total mg/kg dose administered were younger, more likely to come from regions with high relapse periodicity, to be administered primaquine at a higher dose for a shorter duration and to come from the Asia–Pacific region (Additional file 1: Table S9).

**Fig. 3:**
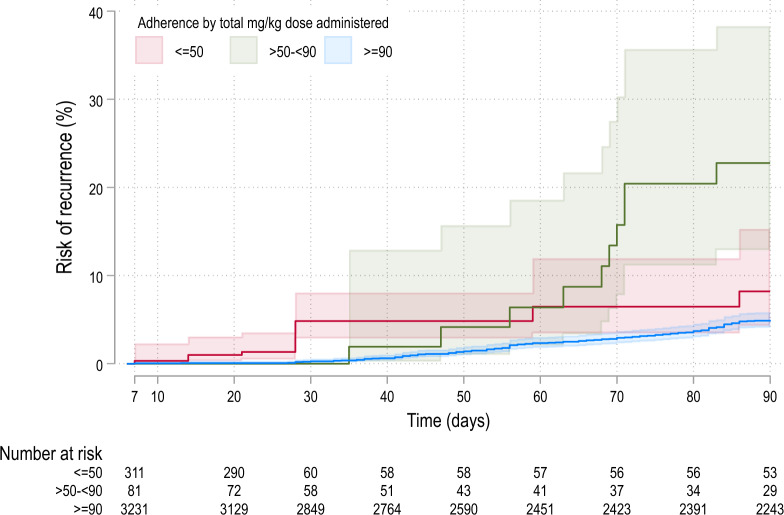
Risk of the first recurrence between days 7 and 90 in patients with adherence by total mg/kg dose administered. The shaded areas represent 95% CIs

Overall, 153 (4.1%) patients had recurrence between days 7 and 90, of whom 16 (10.5%) had ≤ 50% adherence, 10 (6.5%) had > 50- < 90% adherence and 127 (83%) had ≥ 90% adherence. The cumulative risks of recurrence at day 42 were 4.8% [2.8–8.0] in patients with ≤ 50% adherence, 1.9% [0.3–12.8] in patients with > 50- < 90% adherence and 0.8% [0.6–1.3] in patients with ≥ 90% adherence; p < 0.001. The corresponding risks at 90 days were 8.2% [4.3–15.2], 22.7% [12.9–38.3] and 4.9% [4.1–5.8]; p < 0.001 (Fig. 3). In total, 79.4% (247/311) of patients in the ≤ 50% group were censored by day 28 in the analysis, and 73.8% (256/347) of these patients in this group were from a single study conducted in Vietnam which had 28 days follow-up (Additional file 1: Table S5-panel B)) [28].

After controlling for age, sex, and baseline parasitaemia, reduced adherence was significantly associated with an increased rate of recurrence between days 7 to 90: AHR = 7.6 [95% CI 1.9–30.0], p = 0.004, for ≤ 50% compared to ≥ 90% adherence and AHR = 3.2 [95% CI 1.5–6.7], p = 0.003, for > 50- < 90% compared to ≥ 90% adherence (Table 3). As the Kaplan–Meier survival curves crossed at approximately 56 days, the comparative rate of *P. vivax* recurrence was estimated using a time-varying HR before and after this time (Table 3 and Additional file 1: Table S10). In a sensitivity analysis removing one study site at a time, the coefficient of variation for the ≤ 50% adherence group was 18.7%, with AHRs ranging from 2.8 to 10.7. The corresponding figure for the moderate adherence group was 14.1%, and AHRs ranged from 2.5 to 5.1 (Additional file 1: Table S11).

**Table 3:** Effect of the total mg/kg dose administered on the risk of *Plasmodium vivax* recurrence between days 7 and 90 in patients with information on the total mg/kg dose administered

Adherence by total mg/kg dose administered	Overall risk of recurrence between days 7 and 90			Risk of recurrence between days 7 and 56			Risk of recurrence between days 56 and 90		
	Number of recurrences (Number of patients) †	Adjusted HR* (95% CI)	p-value	Number of recurrences (Number of patients)	Adjusted HR* (95% CI)	p-value	Number of recurrences (Number of patients)	Adjusted HR* (95% CI)	p-value
≥ 90%	127 (3231)	Ref.	.	49 (691)	Ref.	.	78 (2540)	Ref.	.
> 50- < 90%	10 (81)	3.2 (1.5–6.7)	0.003	2 (38)	1.11 (0.3–4.8)	0.884	8 (43)	6.0 (2.6–13.9)	< 0.001
≤ 50%	16 (311)	7.6 (1.9–30.0)	0.004	14 (253)	10.1 (1.9–52.5)	0.006	2 (58)	3.3 (0.3–38.6)	0.336

HR: hazard ratio, 95% CI: 95% confidence interval

^*^ Adjusted for age, sex, baseline parasitaemia, and study site as a random effect (a detailed result is presented in Additional file 1: Table S10)

^†^ Data were missing for sex (one patient), and baseline parasitaemia (49 patients)

### Adherence by actual doses of primaquine administered

Among 2910 patients with actual information on doses of primaquine administered, 86 (3.0%) patients had imperfect adherence (< 90%). Due to the low number of patients with imperfect adherence, the risk of recurrence was not assessed by actual dosing (Additional file 1: Table S12 presents demographic and baseline characteristic for adherence by actual dosing groups).

To define the key factors contributing to imperfect adherence according to the actual dose of primaquine administered, the association between patient characteristics and adherence was explored. Primaquine dosing information, patients’ characteristics, and clinical markers that could be associated with imperfect adherence were included. There was a trend to higher odds of imperfect adherence with increased primaquine daily dosing (for every 0.25 mg/kg odds ratio (OR) = 1.1, 95% CI 0.9–1.4; p = 0.228) (Table 4). Expected primaquine duration was not associated with imperfect adherence (OR = 1.2 for < 14 day versus 14-day regimen, 95% CI 0.7–2.2; p = 0.551) (Table 4).

**Table 4:** Risk factors for imperfect adherence (< 90%) in patients with information on actual dose administered

	Adherence by actual dose administered		Univariable analyses	
	≥ 90% (n = 2824)	< 90% (n = 86)	Unadjusted OR (95% CI)	p-value
Primaquine daily dose, every 0.25 mg/kg increase	2824 (97.0%)	86 (3.0%)	1.1 (0.9–1.4)	0.288
Primaquine regimen duration				
14 days	1604 (97.3%)	45 (2.7%)	Ref.	.
<14 days	1220 (96.8%)	41 (3.2%)	1.2 (0.7–2.2)	0.551
Start day of primaquine				
Day 2/3	80 (97. 6%)	2 (2.4%)	Ref.	.
Day 0/1	2744 (97.0%)	84 (3.0%)	1.2 (0.8–1.8)	0.323
Vomiting				
No	1314 (96.6%)	46 (3.4%)	Ref.	.
Yes	518 (97.0%)	16 (3.0%)	0.9 (0.4–2.1)	0.775
Sex				
Male	1815 (96.7%)	63 (3.3%)	Ref	.
Female	1009 (97.8%)	23 (2.2%)	0.7 (0.4–1.0)	0.068
Age (years)	2824 (97.0%)	86 (3.0%)	1.0 (0.98–1.01)	0.821
Age category (years)				
≥ 15	1726 (96.8%)	57 (3.2%)	Ref.	.
5- < 15	930 (97.6%)	23 (2.4%)	0.8 (0.5–1.1)	0.131
< 5	168 (96.6%)	6 (3.4%)	1.1 (0.4–2.9)	0.878
Parasitaemia, parasites per μL every tenfold increase	2768 (97.0%)	85 (3.0%)	1.0 (0.7–1.3)	0.887
Fever at baseline, temperature > 37.5°C				
No	1609 (97.28%)	45 (2.72%)	Ref.	.
Yes	1032 (96.45%)	38 (3.55%)	1.3 (0.8–2.1)	0.246

*OR:* odds ratio

Data were missing for the following variables: baseline parasitaemia (56 patients in ≥ 90% group and one patient in the < 90% group), baseline haemoglobin (61 patients in ≥ 90% group and 2 patient in the < 90% group), weight (54 patients in ≥ 90% group and 2 patient in the < 90% group), fever (183 patients in the ≥ 90% group and 3 patients in < 90%), and vomiting (992 patients in the ≥ 90% group and 24 patients in < 90%)

## Discussion

In this pooled analysis of individual patient data from prospective clinical efficacy studies, poor adherence to primaquine was associated with a greater risk of *P. vivax* recurrence, and this was consistent for different definitions of adherence. Overall, adherence in the context of clinical trials was high, with no factors identified that were associated with imperfect adherence.

Recommended anti-malarial regimens for *P. vivax* include a three-day course of schizontocidal drugs such as chloroquine or artemisinin-based combination therapy, plus a 7- to 14-day course of primaquine for hypnozoitocidal treatment [50]. Adherence to the blood schizontocidal therapy tends to be better when patients are clinically unwell with malaria, however, subsequent adherence to primaquine then decreases as patients begin to feel better [16, 51]. In turn, poor adherence and supervision have been associated with reduced effectiveness of primaquine radical cure regimens [7, 52]. Therefore, measuring adherence to treatment and identifying factors relating to reduced adherence is important [22, 23].

In this study there was a 2.3-fold increase in the risk of recurrence when adherence was $\le$ 50%, when defined according to the level of supervision. These findings are based on clinical efficacy studies with active case follow-up and encouragement for taking medication; thus, patients would be expected to be more adherent than in routine clinical practice. The results are consistent with a 2010 study from the Thailand-Myanmar border, in which patients treated with directly observed therapy were six times less likely to have a recurrence during 90-day follow-up compared to those self-administering therapy [53]. Similarly, in Ethiopia, supervision of primaquine was associated with a fourfold reduction in the risk of recurrence within 12 months [35]. Conversely, a study in Afghan refugees found the anti-relapse efficacy of 14-day primaquine in patients with supervised and unsupervised treatment was similar, suggesting adherence may vary between populations [54]. Previous studies have also shown that when primaquine is administered outside efficacy studies, the impact of reduced adherence may be apparent [6, 7]. Real life data reporting passive detection of malaria from the Brazilian National Malaria Control Programme found a twofold increased risk of recurrence within 180 days with unsupervised versus supervised 7-day primaquine [8] and historical data from American soldiers repatriated from Vietnam found a 4.4-fold increase in the odds of recurrence with unsupervised versus supervised 14-day primaquine [55].

Treatment strategies to overcome non-adherence are now receiving greater attention. Two recent studies demonstrated the non-inferiority of 7-day high dose primaquine compared with 14-day high dose primaquine [41, 47]. In 2019, tafenoquine, a new hypnozoitocidal agent for the anti-relapse treatment of *P. vivax* was licensed [56]. Unlike primaquine, tafenoquine is a single-dose treatment, avoiding the potential concerns around adherence [44]. However, both tafenoquine and 7-day primaquine have potential barriers to implementation due to the inability to cease tafenoquine and prevent haemolysis in G6PD deficient individuals and the potential risk of increased haemolysis in primaquine regimens with a higher daily dose [56].

Although the duration of anti-malarial treatment is a potential determinant of adherence [ 53, 57﻿], the results of this pooled analysis of clinical trial studies did not find an association between the duration of primaquine regimen and adherence. The confounding associated with a higher daily primaquine dose could not be adjusted for due to few patients with imperfect adherence reducing power to undertake multivariable analysis.

Several factors have been linked with the recurrence of vivax malaria and were considered in the imperfect adherence analysis. These factors include younger age, male sex, the level of parasitaemia on admission, fever, and history of malaria [38, 53, 58, 59]. Results of this study suggested male sex, and a higher mg/kg daily primaquine dose may be associated with poor adherence and greater risk of recurrent *P. vivax*, but the confidence intervals for the estimates of these factors were wide and a multivariable analysis was not undertaken due to few patients with imperfect adherence.

This study has several additional limitations. First, the analysis included less than half of the patients from the clinical trials targeted and no studies since March 2020 (due to the time required to receive individual patient data from study investigators and curation in the WWARN repository). However, a sensitivity analysis in which study sites were removed one at a time revealed minimal bias relating to the individual study sites that were included. Moreover, a high proportion of patients (45%) in this pooled analysis were from three studies that include patients recruited from six countries [29, 41, 47] (Additional file 1: Table S5). The baseline characteristics of patients included had similar characteristics to those from studies that were targeted but not available for inclusion in the analysis (Additional file 1: Table S4). Second, the inclusion of clinical efficacy studies with active follow-up of patients and supervised treatment in many cases meant that a high proportion of patients had full adherence based on supervision or total mg/kg dose administered, and an assessment of adherence could not be made based on actual doses administered. Instead, adherence was inferred based on level of supervision or total mg/kg dose administered. This likely reduces the ability to assess some potential determinants of adherence, such as the acceptability profile of a regimen including side effects, pill burden and duration. Third, patients from one study with only 28 days of follow-up [28] composed 74% of the patients with $\le$ 50% adherence by total mg/kg dose. Censoring at 28 days for these patients prevented an understanding of the risk of recurrence beyond this time and likely contributed to the time-varying hazard ratio in the Cox regression model assessing the risk of recurrence over 90 days.

Although recurrent parasitaemia can be due to relapsing malaria caused by failure of effective anti-relapse therapy to kill hypnozoites from the liver, it can also be due to new infections or recrudescence related to ineffective blood stage treatment. A lack of current standardized methodology to distinguish these causes, prevented the categorization of recurrent events in the current analysis. A further limitation of the current study was the lack of assessment of adherence on late relapses which can occur > 6 months after initial infection and cannot be easily distinguished from reinfections. Schizontocidal anti-malarial elimination half-life leads to a variable period of post-treatment prophylaxis, preventing recurrent parasitaemia. Over 95% of patients in the current study were treated with schizontocidal anti-malarials with a long elimination half-life and the distribution of patients across schizontocide elimination half-life categories was similar across adherence groups.

## Conclusions

In summary, this pooled analysis of individual patient data highlights that even in clinical studies with active follow-up, the risk of *P. vivax* recurrence is increased when patients have reduced adherence. These results highlight the need for future studies to look beyond the efficacy of radical cure regimens and evaluate their effectiveness amidst real-world implementations. In addition, mathematical models that aim to predict the impact of anti-relapse regimens need to incorporate estimates of imperfect adherence. Improvements in adherence can be achieved by understanding the determinants of adherence and implementing varied interventions based on the sociocultural contexts of different endemic settings [52, 57]. These findings reinforce the need for national malaria control programmes and researchers to consider alternative regimens and methods to improve adherence when anti-relapse therapy is implemented.

### Supplementary Information

**Additional file 1: Checklist S1**. PRISMA-IPD. **Box S1**. Search Strategy. **Table S1**. Studies included in analysis. **Table S2**. Reasons for studies not being included in analysis. **Table S3**. Studies targeted for the analysis but not included. **Figure S1**. Study sites for clinical trial – Africa Region. **Figure S2**. Study sites for clinical trial – Americas Region. **Figure S3**. Study sites for clinical trial – Asia-Pacific Region. **Table S4**. Comparison of baseline characteristics between included and targeted studies. **Table S5**. Distribution (number and percentage) of patients in adherence categories by study for (A) supervision, (B) total mg/kg dose administered. **Table S6**: Risk factors for *Plasmodium vivax* recurrence between days 7 and 90 in patients with information on supervision. **Table S7**. Sensitivity analyses for effect of adherence by supervision on *Plasmodium vivax* recurrence between days 7 to 90 restricted to randomised studies or observational studies. **Table S8**. Sensitivity analysis for effect of adherence by supervision on *Plasmodium vivax* recurrence between days 7 to 90. **Figure S4**: Adjusted risk of recurrence between days 7 and 90 in patients with information on supervision. **Table S9**: Demographic and baseline characteristics for adherence by total mg/kg dose administered. **Table S10**. Risk factors for *Plasmodium vivax* recurrence between days 7 and 90 in patients with information on total mg/kg dose administered. **Table S11**. Sensitivity analysis for effect of adherence by total dose (mg/kg) administered on *P. vivax* recurrence between days 7 to 90. **Table S12**: Demographic and baseline characteristics for adherence by actual dosing. **References S1**: Studies not included in analysis.

## Data Availability

The data are available for access via the WorldWide Antimalarial Resistance Network (WWARN.org). Requests for access will be reviewed by a Data Access Committee to ensure that use of data protects the interests of the participants and researchers according to the terms of ethics approval and principles of equitable data sharing. Requests can be submitted by email to malariaDAC@iddo.org via the Data Access Form available at WWARN.org/accessing-data. The WWARN is registered with the Registry of Research Data Repositories (re3data.org).

## Abbreviations

PROSPERO: International Prospective Register of Systematic Reviews
WWARN: WorldWide Antimalarial Resistance Network
AHR: Adjusted Hazard ratio
IQR: Interquartile Range
CI: Confidence Interval
