# Supplementary material for: Effect of adherence to primaquine on the risk of Plasmodium vivax recurrence: a WorldWide Antimalarial Resistance Network systematic review and individual patient data meta-analysis
Source: Malar J. 2023 Oct 10;22:306. doi: 10.1186/s12936-023-04725-w (PMC10563365; doi:10.1186/s12936-023-04725-w)
Supplement: Supplementary file 1 — Additional file 1: Checklist S1. PRISMA-IPD. Box S1. Search Strategy. Table S1. Studies included in analysis. Table S2. Reasons for studies not being included in analysis. Table S3. Studies targeted for the analysis but not included. Figure S1. Study sites for clinical trial – Africa Region. Figure S2. Study sites for clinical trial – Americas Region. Figure S3. Study sites for clinical trial – Asia-Pacific Region. Table S4. Comparison of baseline characteristics between included and targeted studies. Table S5. Distribution (number and percentage) of patients in adherence categories by study for (A) supervision, (B) total mg/kg dose administered. Table S6: Risk factors for Plasmodium vivax recurrence between days 7 and 90 in patients with information on supervision. Table S7. Sensitivity analyses for effect of adherence by supervision on Plasmodium vivax recurrence between days 7 to 90 restricted to randomised studies or observational studies. Table S8. Sensitivity analysis for effect of adherence by supervision on Plasmodium vivax recurrence between days 7 to 90. Figure S4: Adjusted risk of recurrence between days 7 and 90 in patients with information on supervision. Table S9: Demographic and baseline characteristics for adherence by total mg/kg dose administered. Table S10. Risk factors for Plasmodium vivax recurrence between days 7 and 90 in patients with information on total mg/kg dose administered. Table S11. Sensitivity analysis for effect of adherence by total dose (mg/kg) administered on P. vivax recurrence between days 7 to 90. Table S12: Demographic and baseline characteristics for adherence by actual dosing. References S1: Studies not included in analysis. [file 12936_2023_4725_MOESM1_ESM.pdf]

SUPPLEMENTARY FILE

Mehdipour P, *et al*, Effect of adherence to primaquine on the risk of *Plasmodium vivax* recurrence: A WorldWide Antimalarial Resistance Network systematic review and individual patient data meta-analysis

Outline	Page
**Checklist S1.** PRISMA-IPD	2-5
**Box S1.** Search Strategy	6
**Table S1.** Studies included in analysis	7
**Table S2.** Reasons for studies not being included in analysis	9
**Table S3.** Studies targeted for the analysis but not included	10
**Figure S1.** Study sites for clinical trial – Africa Region	13
**Figure S2.** Study sites for clinical trial – Americas Region	14
**Figure S3.** Study sites for clinical trial – Asia-Pacific Region	15
**Table S4.** Comparison of baseline characteristics between included and targeted studies	16
**Table S5.** Distribution (number and percentage) of patients in adherence categories by study for (A) supervision, (B) total mg/kg dose administered	17-18
**Table S6:** Risk factors for *Plasmodium vivax* recurrence between days 7 and 90 in patients with information on supervision	19
**Table S7.** Sensitivity analyses for effect of adherence by supervision on *Plasmodium vivax* recurrence between days 7 to 90 restricted to randomised studies or observational studies	20
**Table S8.** Sensitivity analysis for effect of adherence by supervision on *Plasmodium vivax* recurrence between days 7 to 90	20
**Figure S4:** Adjusted risk of recurrence between days 7 and 90 in patients with information on supervision	21
**Table S9:** Demographic and baseline characteristics for adherence by total mg/kg dose administered	22
**Table S10.** Risk factors for *Plasmodium vivax* recurrence between days 7 and 90 in patients with information on total mg/kg dose administered	23
**Table S11.** Sensitivity analysis for effect of adherence by total dose (mg/kg) administered on *P. vivax* recurrence between days 7 to 90	25
**Table S12:** Demographic and baseline characteristics for adherence by actual dosing	26
**References S1.** Studies not included in analysis	27

Checklist S1. PRISMA-IPD Checklist of items to include when reporting a systematic review and meta-analysis of individual participant data (IPD)

PRISMA-IPD Section/topic	Item No	Checklist item	Reported on page
**Title**			
Title	1	Identify the report as a systematic review and meta-analysis of individual participant data.	1
**Abstract**			
Structured summary	2	Provide a structured summary including as applicable:	7-8
		**Background**: state research question and main objectives, with information on participants, interventions, comparators and outcomes.	
		**Methods**: report eligibility criteria; data sources including dates of last bibliographic search or elicitation, noting that IPD were sought; methods of assessing risk of bias.	
		**Results**: provide number and type of studies and participants identified and number (%) obtained; summary effect estimates for main outcomes (benefits and harms) with confidence intervals and measures of statistical heterogeneity. Describe the direction and size of summary effects in terms meaningful to those who would put findings into practice.	
		**Discussion:** state main strengths and limitations of the evidence, general interpretation of the results and any important implications.	
		**Other:** report primary funding source, registration number and registry name for the systematic review and IPD meta-analysis.	
**Introduction**			
Rationale	3	Describe the rationale for the review in the context of what is already known.	9
Objectives	4	Provide an explicit statement of the questions being addressed with reference, as applicable, to participants, interventions, comparisons, outcomes and study design (PICOS). Include any hypotheses that relate to particular types of participant-level subgroups.	9
**Methods**			
Protocol and registration	5	Indicate if a protocol exists and where it can be accessed. If available, provide registration information including registration number and registry name. Provide publication details, if applicable.	10
Eligibility criteria	6	Specify inclusion and exclusion criteria including those relating to participants, interventions, comparisons, outcomes, study design and characteristics (e.g. years when conducted, required minimum follow-up). Note whether these were applied at the study or individual level i.e. whether eligible participants were included (and ineligible participants excluded) from a study that included a wider population than specified by the review inclusion criteria. The rationale for criteria should be stated.	10
Identifying studies - information sources	7	Describe all methods of identifying published and unpublished studies including, as applicable: which bibliographic databases were searched with dates of coverage; details of any hand searching including of conference proceedings; use of study registers and agency or company databases; contact with the original research team and experts in the field; open adverts and surveys. Give the date of last search or elicitation.	10-12
Identifying studies - search	8	Present the full electronic search strategy for at least one database, including any limits used, such that it could be repeated.	10 and Appendix

Study selection processes	9	State the process for determining which studies were eligible for inclusion.	10
Data collection processes	10	Describe how IPD were requested, collected and managed, including any processes for querying and confirming data with investigators. If IPD were not sought from any eligible study, the reason for this should be stated (for each such study).	10-12
		If applicable, describe how any studies for which IPD were not available were dealt with. This should include whether, how and what aggregate data were sought or extracted from study reports and publications (such as extracting data independently in duplicate) and any processes for obtaining and confirming these data with investigators.	
Data items	11	Describe how the information and variables to be collected were chosen. List and define all study level and participant level data that were sought, including baseline and follow-up information. If applicable, describe methods of standardising or translating variables within the IPD datasets to ensure common scales or measurements across studies.	10-11
IPD integrity	A1	Describe what aspects of IPD were subject to data checking (such as sequence generation, data consistency and completeness, baseline imbalance) and how this was done.	10
Risk of bias assessment in individual studies.	12	Describe methods used to assess risk of bias in the individual studies and whether this was applied separately for each outcome. If applicable, describe how findings of IPD checking were used to inform the assessment. Report if and how risk of bias assessment was used in any data synthesis.	Appendix
Specification of outcomes and effect measures	13	State all treatment comparisons of interests. State all outcomes addressed and define them in detail. State whether they were pre-specified for the review and, if applicable, whether they were primary/main or secondary/additional outcomes. Give the principal measures of effect (such as risk ratio, hazard ratio, difference in means) used for each outcome.	12
Synthesis methods	14	Describe the meta-analysis methods used to synthesise IPD. Specify any statistical methods and models used. Issues should include (but are not restricted to):<br>• Use of a one-stage or two-stage approach.<br>• How effect estimates were generated separately within each study and combined across studies (where applicable).<br>• Specification of one-stage models (where applicable) including how clustering of patients within studies was accounted for.<br>• Use of fixed or random effects models and any other model assumptions, such as proportional hazards.<br>• How (summary) survival curves were generated (where applicable).<br>• Methods for quantifying statistical heterogeneity (such as I^2 and τ^2).<br>• How studies providing IPD and not providing IPD were analysed together (where applicable).<br>• How missing data within the IPD were dealt with (where applicable).	13
Exploration of variation in effects	A2	If applicable, describe any methods used to explore variation in effects by study or participant level characteristics (such as estimation of interactions between effect and covariates). State all participant-level characteristics that were analysed as potential effect modifiers, and whether these were pre-specified.	14
Risk of bias across studies	15	Specify any assessment of risk of bias relating to the accumulated body of evidence, including any pertaining to not obtaining IPD for particular studies, outcomes or other variables.	Appendix

Additional analyses	16	Describe methods of any additional analyses, including sensitivity analyses. State which of these were pre-specified.	14
**Results**			
Study selection and IPD obtained	17	Give numbers of studies screened, assessed for eligibility, and included in the systematic review with reasons for exclusions at each stage. Indicate the number of studies and participants for which IPD were sought and for which IPD were obtained. For those studies where IPD were not available, give the numbers of studies and participants for which aggregate data were available. Report reasons for non-availability of IPD. Include a flow diagram.	15
Study characteristics	18	For each study, present information on key study and participant characteristics (such as description of interventions, numbers of participants, demographic data, unavailability of outcomes, funding source, and if applicable duration of follow-up). Provide (main) citations for each study. Where applicable, also report similar study characteristics for any studies not providing IPD.	15-16
IPD integrity	A3	Report any important issues identified in checking IPD or state that there were none.	15-18 and Appendix
Risk of bias within studies	19	Present data on risk of bias assessments. If applicable, describe whether data checking led to the up-weighting or down-weighting of these assessments. Consider how any potential bias impacts on the robustness of meta-analysis conclusions.	Appendix
Results of individual studies	20	For each comparison and for each main outcome (benefit or harm), for each individual study report the number of eligible participants for which data were obtained and show simple summary data for each intervention group (including, where applicable, the number of events), effect estimates and confidence intervals. These may be tabulated or included on a forest plot.	15-18
Results of syntheses	21	Present summary effects for each meta-analysis undertaken, including confidence intervals and measures of statistical heterogeneity. State whether the analysis was pre-specified, and report the numbers of studies and participants and, where applicable, the number of events on which it is based.	15-18
		When exploring variation in effects due to patient or study characteristics, present summary interaction estimates for each characteristic examined, including confidence intervals and measures of statistical heterogeneity. State whether the analysis was pre-specified. State whether any interaction is consistent across trials.	
		Provide a description of the direction and size of effect in terms meaningful to those who would put findings into practice.	
Risk of bias across studies	22	Present results of any assessment of risk of bias relating to the accumulated body of evidence, including any pertaining to the availability and representativeness of available studies, outcomes or other variables.	Appendix
Additional analyses	23	Give results of any additional analyses (e.g. sensitivity analyses). If applicable, this should also include any analyses that incorporate aggregate data for studies that do not have IPD. If applicable, summarise the main meta-analysis results following the inclusion or exclusion of studies for which IPD were not available.	18
**Discussion**			

Summary of evidence	24	Summarise the main findings, including the strength of evidence for each main outcome.	19
Strengths and limitations	25	Discuss any important strengths and limitations of the evidence including the benefits of access to IPD and any limitations arising from IPD that were not available.	21
Conclusions	26	Provide a general interpretation of the findings in the context of other evidence.	22
Implications	A4	Consider relevance to key groups (such as policy makers, service providers and service users). Consider implications for future research.	20-21
**Funding**			
Funding	27	Describe sources of funding and other support (such as supply of IPD), and the role in the systematic review of those providing such support.	24

© Reproduced with permission of the PRISMA IPD Group, which encourages sharing and reuse for non-commercial purposes

Box S1. Search strategy

Search strategy

In order to collect data, we updated a comprehensive systematic review undertaken in 2017 [10] of all the prospective *P. vivax* clinical efficacy studies. We searched MEDLINE, Web of Science, Embase, and Cochrane Database of Systematic Reviews, according to the Preferred Reporting Items for Systematic Reviews and Meta-Analyses statement from Jan 1, 1999, to March 3, 2020, in any language. In the analysis, we included prospective therapeutic efficacy studies including randomized and non-randomized therapeutic trials and prospective cohort studies with active follow-up. Studies on prevention, prophylaxis, reviews, animal studies, patients with severe malaria were excluded. Prospective clinical efficacy studies of uncomplicated vivax malaria with a minimum of 28 days of follow-up, daily primaquine (commenced before day 3) administered with schizontocidal treatments – chloroquine or artemisinin-based combination therapy, information on schizontocidal treatment dosing, supervision, timing, and dose of primaquine administered and planned per study protocol were included. The year of the study was taken as the year in which the paper was published, although the start and end date of patient enrolment were also recorded. Two independent investigators performed the review and identified relevant studies, resolving discrepancy through discussion. Principal investigators of eligible studies were invited to share individual patient data and any additional data from eligible unpublished studies.

Key terms

A literature search (conducted March 3, 2020) with the following key terms was performed (version undertaken in Pubmed): Vivax AND (artefenomel OR arterolane OR amodiaquine OR atovaquone OR artemisinin OR arteether OR artesunate OR artemether OR artemotil OR azithromycin OR artekin OR chloroquine OR chlorproguanil OR cycloguanil OR clindamycin OR coartem OR dapsone OR dihydroartemisinin OR duo-cotecxin OR doxycycline OR halofantrine OR lumefantrine OR lariam OR malarone OR mefloquine OR naphthoquine OR naphthoquinone OR piperaquine OR primaquine OR proguanil OR pyrimethamine OR pyronaridine OR proguanil OR quinidine OR quinine OR riamet OR sulphadoxine OR tetracycline OR tafenoquine).

Table S1. Studies included in analysis

Author-year	Country	Recruitment Period	Age groups (years) <5	5-15	>15	Follow up (days)	Treatment arms*	Primaquine information: Start day	Duration	Total dose	Total patients in study	Patients included in study	G6PD activity study inclusion criteria	Adherence data in study: Super vision	Actual dosing	Total mg/kg dose
Hasugian-2007 [19]	Indonesia	2005	Yes	Yes	Yes	84	AsAq_Pq_4.2_14d_D2, Dp_Pq_4.2_14d_D2	2, 2	14d, 14d	4.2, 4.2	340	115	≥30% activity	Yes	No	No
Leslie-2008 [20]	Pakistan	2004 - 2006	Yes	Yes	Yes	330	Cq, Cq_Pq_7.0_14d_D0	NA, 0	NA, 14d	NA, 7	210	125	All activities (if deficient given weekly PQ)	Yes	No	No
Abdallah-2012 [21]	Sudan	2011	Yes	Yes	Yes	28	Al_Pq_4.2_14d_D0	0	14d	4.2	38	37	All activities (G6PD testing was not available)	Yes	No	No
Llanos-Cuentas-2014 [49]	Multinational	2010 - 2013	No	No	Yes	180	Cq, Cq_Pq_3.5_14d_D1	NA, 1	NA, 14d	NA, 3.5	424	103	≥70% activity	Yes	No	No
Marques-2014 [22]	Brazil	2007 - 2008	No	Yes	Yes	28	Cq_Pq_3.5_7-9d_D0	0	7-9d	3.5	154	135	Not stated	Yes	No	No
Gomes-2015 [23]	Brazil	2011	No	Yes	Yes	28	Cq_Pq_3.5_7-12d_D0	0	7-12d	3.5	94	93	Not stated	Yes	No	Yes
Gonzalez-Ceron-2015 [24]	Mexico	2008 - 2010	Yes	Yes	Yes	365	Cq_Pq_3.5_14d_D0	0	14d	3.5	159	88	All activities (if deficient given weekly PQ)	Yes	No	Yes
Lidia-2015 [25]	Indonesia	2013	No	No	Yes	42	Cq_Pq_3.5_14d_D0, Dp_Pq_3.5_14d_D0	0, 0	14d, 14d	3.5, 3.5	51	51	Not stated	Yes	No	No
Nelwan-2015 [26]	Indonesia	2013	No	No	Yes	365	Dp_Pq_7.0_14d_D0	0	14d	7	180	56	≥30% activity	Yes	Yes	Yes
Rishikesh-2015 [27]	India	2012 - 2015	No	No	Yes	28	Cq_Pq_3.5_14d_D2	2	14d	3.5	125	117	≥30% activity	Yes	No	No
Thanh-2015 [28]	Vietnam	2009 - 2010	Yes	Yes	Yes	28	Cq_Pq_5.0_10d_D0	0	10d	5	260	260	All activities (if deficient not given PQ)	Yes	No	Yes
Yuan-2015 [29]	Myanmar	2012 - 2013	Yes	Yes	Yes	42	Cq_Pq_3.0_8d_D0	0	8d	3	594	588	No screening undertaken	Yes	No	No
Ley-2016 [30]	Bangladesh	2014 - 2015	Yes	Yes	Yes	30	Cq_Pq_3.5_14d_D2	2	14d	3.5	181	55	All activities (if deficient not given PQ)	Yes	No	Yes
Longley-2016 [31]	Thailand	2014	No	Yes	Yes	270	Cq_Pq_3.5_14d_D1	1	14d	3.5	57	43	All activities (if deficient not given PQ)	Yes	Yes	Yes
Pereira-2016 [32]	Brazil	2013 - 2014	No	No	Yes	28	Cq_Pq_3.5_7d_D0, Cq_Pq_4.0_8d_D0, Cq_Pq_4.5_9d_D0	0, 0, 0	7d, 8d, 9d	3.5, 4, 4.5	88	86	No prior known G6PD deficiency	Yes	No	No
Saravu-2016 [33]	India	2012 - 2015	No	No	Yes	28	Cq_Pq_3.5_14d_D0	0	14d	3.5	161	156	All activities (if deficient given weekly PQ)	Yes	No	No
Zuluaga-Idarraga-2016 [34]	Colombia	2012 - 2013	Yes	Yes	Yes	180	Cq_Pq_3.5_14d_D0	0	14d	3.5	87	87	Not stated	Yes	No	Yes
Abreha-2017 [35]	Ethiopia	2012 - 2014	Yes	Yes	Yes	365	Al, Al_Pq_3.5_14d_D2, Cq, Cq_Pq_3.5_14d_D2	NA, 2, NA, 2	NA, 14d, NA, 14d	NA, 3.5, NA, 3.5	399	397	≥30% activity	Yes	No	No
Awab-2017 [36]	Afghanistan	2009 - 2013	Yes	Yes	Yes	390	Cq, Cq_Pq_3.5_14d_D0	NA, 0	NA, 14d	NA, 3.5	570	544	≥30% activity	Yes	No	No
Brasil-2018 [37]	Brazil	2012 - 2014	Yes	Yes	Yes	180	Cq_Pq_3.5_7d_D0	0	7d	3.5	190	190	Not stated	Yes	No	No
Chu-2018 [38]	Thailand	2010 - 2011	Yes	Yes	Yes	365	Cq, Cq_Pq_7.0_14d_D0	NA, 0	NA, 14d	NA, 7	655	420	All activities (if deficient not given PQ)	Yes	Yes	Yes

**Daher-2018** [39]	Brazil	2012 - 2015	No	No	Yes	63	Al_Pq_3.5_7-9d_D0, AsMf_Pq_3.5_7-9d_D0, Cq_Pq_3.5_7-9d_D0	0, 0, 0	7-9d, 7-9d, 7-9d	3.5, 3.5, 3.5	264	264	All activities (G6PD testing was not available)	Yes	No	No
**Saravu-2018** [40]	India	2017	No	No	Yes	180	Cq_Pq_3.5_14d_D0, Cq_Pq_7.0_14d_D0	0, 0	14d, 14d	3.5, 7	50	50	All activities (if deficient not given PQ)	Yes	No	No
**Chu-2019** [41]	Thailand	2012 - 2014	Yes	Yes	Yes	365	Cq_Pq_7.0_14d_D0, Cq_Pq_7.0_7d_D0, Dp_Pq_7.0_14d_D0, Dp_Pq_7.0_7d_D0	0, 0, 0, 0	14d, 7d, 14d, 7d	7, 7, 7, 7	654	654	≥30% activity	Yes	Yes	Yes
**de Sena-2019** [42]	Brazil	2016 - 2017	Yes	Yes	No	42	Cq_Pq_3.5_7d_D0	0	7d	3.5	113	113	Not stated	Yes	No	Yes
**Lacerda-2019** [45]	Multinational	2013 - 2017	No	No	Yes	180	Cq, Cq_Pq_3.5_14d_D1	NA, 1	NA, 14d	NA, 3.5	683	262	≥70% activity	Yes	No	No
**Ladeia-Andrade-2019** [43]	Brazil	2014 - 2015	No	Yes	Yes	180	Cq_Pq_3.5_7d_D0	0	7d	3.5	204	94	≥30% activity	Yes	No	Yes
**Llanos-Cuentas-2019** [44]	Multinational	2014 - 2017	No	No	Yes	180	Cq_Pq_3.5_14d_D1	1	14d	3.5	369	85	≥70% activity	Yes	No	No
**Rijal-2019** [46]	Nepal	2015 - 2016	No	Yes	Yes	365	Cq, Cq_Pq_3.5_14d_D0	NA, 0	NA, 14d	NA, 3.5	206	206	≥30% activity	Yes	No	No
**Taylor-2019** [47]	Multinational	2014 - 2017	Yes	Yes	Yes	365	Cq, Cq_Pq_7.0_14d_D0, Cq_Pq_7.0_7d_D0, Dp, Dp_Pq_7.0_14d_D0, Dp_Pq_7.0_7d_D0	NA, 0, 0, NA, 0, 0	NA, 14d, 7d, NA, 14d, 7d	NA, 7, 7, NA, 7, 7	2388	2288	≥30% activity	Yes	Yes	Yes
**Xu-2020** [48]	Myanmar	2014 - 2016	Yes	Yes	Yes	28	Cq_Pq_3.5_14d_D0	0	14d	3.5	281	278	All activities (G6PD testing was not available)	Yes	No	No
**Karunajeewa-unpublished**	Vanuatu	2013	Yes	Yes	Yes	84	Al, Al_Pq_3.5_14d_D0, Al_Pq_7.0_14d_D0	NA, 0, 0	NA, 14d, 14d	NA, 3.5, 7	34	26	≥30% activity	Yes	Yes	Yes

ACT – Artemisinin-based combination treatment; As – artesunate; Al – artemether-lumefantrine; Aq-Amodiaquine; Cq – chloroquine; Dp – dihydroartemisinin-piperaquine; Mf-Mefloquine; NA – not applicable; Pq – primaquine.

* Treatment arm codes as treatment1+ treatment2+ Pq mg/kg total dose+ Pq duration (days)+ start day of Pq.

Table S2. Reasons for studies not being included in analysis

Reason	Number of studies	Studies*
Data not available	4	[60–63]
Investigators unable to be contacted	6	[64–69]
Missing minimum data for inclusion	3	[70–72]
No response from investigators	35	[73–107]
Patient data available but no adherence information	3	[108–110]

* References of studies not included in the analysis are provided in References S1

Table S3. Studies targeted for the analysis but not included

First Author	Treatment Arms	Number of Sites	Region	Country	Follow up (days)	Randomised	Recruitment period	Treatment arms*	Pv patients enrolled	Enrolled and treated with PQ	Female (%)	Mean Age (SD)	Median Age (range)
**Rowland-1999** [73]	4	1	Asia-Pacific	Pakistan	28	Yes	1996-1998	Cq_Pq_3.5_14d_DX; Cq_Pq_31.3_5d_DX	700	700	52.5	11.2(-)	Not stated
**Pukrittayakamee-2000** [94]	9	1	Asia-Pacific	Thailand	28	Yes	1992-1998	Cq_Pq_14d_D3; Cq; Pq_3.5_14d_D0; Qu; Mfq; Halo; AS; Am; SP	207	30	0	25 (9)	Not stated
**Bergonzoli-2000** [66]	4	2	Americas	Costa Rica	180	Yes	1994	Cq_Pq_3.5_14d_D0; Cq_Pq_2.75_9d_D0; Cq_Pq_2.0_5d_D0; Cq_Pq_0.75_1d_D0	132	132	Not stated	30.5 (-)	Not stated
**Villalobos-Salcedo-2000** [77]	2	1	Americas	Brazil	28	Yes	1998	Cq_Pq_3.5_14d_D1; Cq_Pq_3.5_14d_D1	79	79	21.5	31.7 (-)	Not stated
**Abdon-2001** [78]	3	1	Americas	Brazil	180	Yes	1994-1995	Cq_Pq_3.5_14d_D0; Cq_Pq_3.5_7d_D0; Cq_Pq_2.5_5d_D0	120	120	37.5	27.3 (-)	Not stated
**Dua-2001** [79]	1	4	Asia-Pacific	India	540	No	1987-2000	Cq_Pq_1.25_5d_D2	5541	5541	Not stated	Not stated	Not stated
**Duarte-2001** [80]	1	1	Americas	Brazil	180	No	1997-1998	Cq_Pq_3.5_14d_D2	50	50	24	31.8 (12.8)	Not stated
**Buchachart-2001** [95]	1	1	Asia-Pacific	Thailand	28	No	1992-1997	Cq_Pq_3.5_4d_D3	593	593	37.1	25 (-)	Not stated
**Yadav-2002** [81]	2	1	Asia-Pacific	India	365	Yes	1988-1991	Cq; Cq_Pq_1.25_5d_D2	1482	759	Not stated	Not stated	Not stated
**Mohapatra-2002** [69]	1	1	Asia-Pacific	India	365	No	1998-2000	Cq_Pq_3.5_14d_DX	110	110	36.4	Not stated	Not stated
**Pinto-2003** [83]	2	1	Americas	Brazil	28	Yes	1997-1998	Cq_Pq_3.5_7d_D2; Cq_Pq_2.5_5d_D2	132	132	37.9	30.7 (-)	Not stated
**da Silva-2003** [64]	8	1	Americas	Brazil	180	Yes	Not stated	Cq_Pq_3.5_7d_D2; Cq_Pq_2.5_5d_D2; As_Pq_3.5_7d_D2; As_Pq_3.5_7d_D2; As_Pq_3.5_7d_D2; As_Pq_2.5_5d_D2; As_Pq_2.5_5d_D2; As_Pq_2.5_5d_D2	240	240	23.3	32.9 (-)	Not stated
**Machado-2003** [82]	1	1	Americas	Brazil	28	No	Not stated	Cq_Pq_3.5_14d_D3	30	30	Not stated	Not stated	Not stated
**Leslie-2004** [72]	3	1	Asia-Pacific	Pakistan	270	Yes	2000	Cq_Pq_3.5_14d_D0; Cq_Pq_3.5_14d_D0	595	-	50.7	12.9 (-)	Not stated
**Hapuarachchi-2004** [84]	1	1	Asia-Pacific	Sri Lanka	28	No	2002	Cq_Pq_3.0_14d_DX	42	35	0	Not stated	Not stated
**Tasanor-2006** [96]	2	1	Asia-Pacific	Thailand	28	Yes	2002-2004	Cq_Pq_3.5_14d_D0; Qu_Pq_3.5_14d_DX	62	62	41.9	Not stated	22
**Krudsood-2006** [68]	2	1	Asia-Pacific	Thailand	28	Yes	2004-2005	Cq_Pq_3.5_7d_D3	141	141	74.5	25.0 (6.7)	Not stated

Alvarez-2006 [71]	3	2	Americas	Colombia	180	Yes	2001	Cq_Pq_0.75_3d_D1; Cq_Pq_1.75_7d_D1; Cq_Pq_3.5_14d_D1	210	210	33	30.1 (12.8)	Not stated
Maguire-2006 [85]	2	1	Asia-Pacific	Indonesia	28	Yes	1996-1999	Cq_Pq_3.5_14d_DX; Mfq_Pq_3.5_14d_DX	243	243	32.9	22.8(-)	Not stated
Krudsood-2007 [97]	2	1	Asia-Pacific	Thailand	28	Yes	2004-2005	Cq_Pq_3.5_14d_D3; Al_Pq_3.5_14d_D3	98	98	28.6	24.3 (6.3)	Not stated
Orjuela-Sanchez-2009 [70]	2	1	Americas	Brazil	336	No	2004-2007	Cq_Pq_3.5_7d_D0; Cq_Pq_3.5_7d_D0	164	164	Not stated	Not stated	Not stated
Carmona-Fonseca-2009 [61]	4	2	Americas	Colombia	120	Yes	2001-2003	Cq_Pq_3.5_14d_D1; Cq_Pq_1.75_3d_D1; Cq_Pq_2.5_3d_D1; Cq_Pq_3.5_3d_D1	188	188	30.4	Not stated	Not stated
Lee-2009b [67]	1	1	Asia-Pacific	Republic of Korea	28	No	2007	Cq_Pq_3.0_14d_D3	142	142	0	Not stated	21 (19-50)
Carmona-Fonseca-2010 [62]	2	1	Americas	Colombia	120	Yes	2005-2008	Cq_Pq_3.5_7d_D1; Cq_Pq_3.5_3d_D1	79	79	Not stated	Not stated	Not stated
Daneshvar-2010 [102]	1	1	Asia-Pacific	Malaysia	28	No	-2007	Cq_Pq_3.5_14d_D1	23	23	0	38.5 (7.6)	Not stated
Takeuchi-2010 [86]	2	1	Asia-Pacific	Thailand	90	Yes	2007-2009	Cq_Pq_3.5_14d_D3; Cq_Pq_3.5_14d_D3	216	216	39.8	Not stated	Not stated
Yeshiwondim-2010 [87]	2	1	Africa	Ethiopia	28	Yes	2003	Cq_Pq_3.5_14d_DX; Cq_Pq_3.5_14d_DX	290	277	45.9	23 (+/-12)	20 (4-60)
Muhamad-2011 [88]	1	1	Asia-Pacific	Thailand	42	No	2008-2009	Cq_Pq_3.5_14d_D0	130	130	50.8	Not stated	22
Maneeboonyang-2011 [97]	2	1	Asia-Pacific	Thailand	90	Yes	2005-2006	Cq_Pq_3.5_14d_D3; Cq_Pq_3.5_14d_D3	92	92	40	Not stated	Not stated
Van Den Eede-2011 [103]	1	1	Americas	Peru	365	No	2008	Cq_Pq_3.5_7d_D0	51	51	49	Not stated	15 (2-80)
Graf-2012 [89]	3	1	Americas	Peru	210	Yes	2005-2008	Cq_Pq_2.5_5d_D0; Cq_Pq_3.5_7d_D0; Cq_Pq_3.5_14d_D0	540	540	Not stated	Not stated	Not stated
Pedro-2012 [90]	1	1	Americas	Brazil	28	No	2005-2011	Cq_Pq_3.5_Xd_DX	47	47	24.5	Not stated	Not stated
Eibach-2012 [65]	1	2	Americas	Guyana	28	No	2009-2010	Al_Pq_3.5_14d_DX	74	74	9.5		24 (5-57)
Zhu-2013 [91]	1	1	Asia-Pacific	China	28	No	2008-2009	Cq_Pq_4_8d_D0	39	39	42.1	43 (-)	Not stated
Liu-2013 [99]	2	1	Asia-Pacific	China	365	Yes	2009-2010	Cq_Pq_4.0_8d_D0; Anq	260	132	14	Not stated	Not stated
Ganguly-2013 [98]	2	1	Asia-Pacific	India	42	Yes	2011-2012	Cq; Cq_Pq_3.5_14d_D0	250	125	10.8	25.2 (-)	Not stated
Macareo-2013 [100]	2	1	Asia-Pacific	Thailand	90	Yes	Not stated	Cq_Pq_7.0_14d_D0; Cq_Tnd	20	-	Not stated	Not stated	Not stated
Delgado-Ratto-2014 [92]	1	1	Americas	Peru	720	No	2008	Cq_Pq_3.5_7d_D0	37	37	48.6	Not stated	15
Cheoymang-2015 [93]	1	1	Asia-Pacific	Thailand	42	No	2008-2009	Cq_Pq_3.5_14d_D1	85	85	34.1	Not stated	Not stated
Pareek-2015 [101]	3	8	Asia-Pacific	India	180	Yes	Not stated	Cq_Pq_3.5_14d_D3; Cq_Pq_3.5_14d_D3; Cq_Pq_3.5_7d_D3	358	358	17.3	Not stated	20

**Negreiros-2016** [104]	1	1	Americas	Brazil	168	No	2014	Cq_Pq_3.5_7d_D0	119	119	45.4	Not stated	23.4 (5-67.3)
**Valecha-2016** [63]	2	9	Asia-Pacific	India	42	Yes	2011-2013	Cq_Pq_3.5_14d_D3; AtmPip_Pq_3.5_14d_D3	317	317	8.2	33.7 (13.5)	Not stated
**Mac Donald-Ottevanger-2017** [74]	2	2	Americas	Suriname	365	Yes	2006-2008	Cq_Pq_3.5_7d_D3; Cq_Pq_3.5_14d_D3	79	79	34.4	Not stated	24.64 (-)
**Dharmawardena-2017** [60]	1	1	Asia-Pacific	Sri Lanka	365	No	2015-2016	Cq_Pq_3.5_14d_D2	32	32	6.8	Not stated	35.5 (13-66)
**Fukuda-2017** [105]	2	1	Asia-Pacific	Thailand	120	Yes	2003-2005	Cq_Pq_3.5_14d_D0; Cq_Pq_3.5_14d_D3	70	24	17	Not stated	30 (20-55)
**Mesa-Echeverry-2019** [75]	1	2	Americas	Colombia	28	No	2012-2013	Cq_Pq_3.5_14d_D0	77	77	48.1	25.8 (14.4)	22.0 (-)
**Pham-2019** [106]	1	4	Asia-Pacific	Vietnam	730	No	2009-2011	Cq_Pq_5.0_10d_D0	260	260	39	Not stated	Not stated
**Han-2020** [76]	1	4	Asia-Pacific	Myanmar	28	No	2017-2019	Pyn-AS_Pq_14d_DX	206	206	34.9	27.2 (13.9)	Not stated

Al – artemether-lumefantrine; Am - artemether; ART - artemisinin; As – artesunate; Atm – arterolane maleate; Bq – bulaquine; Cq – chloroquine; Halo – halofantrine; Mfq – mefloquine; Nq - naphthoquine; Pip – piperaquine; Pq – primaquine; Pyn Pyronaridine; Qu – Quinine; SD – standard deviation; SP – sulfadoxine-pyrimethamine; Tnd – tinidazole; Tq – tafenoquine.

* Treatment arms in study described as drug, number of days given and start day of Pq if Cq+/-Pq.

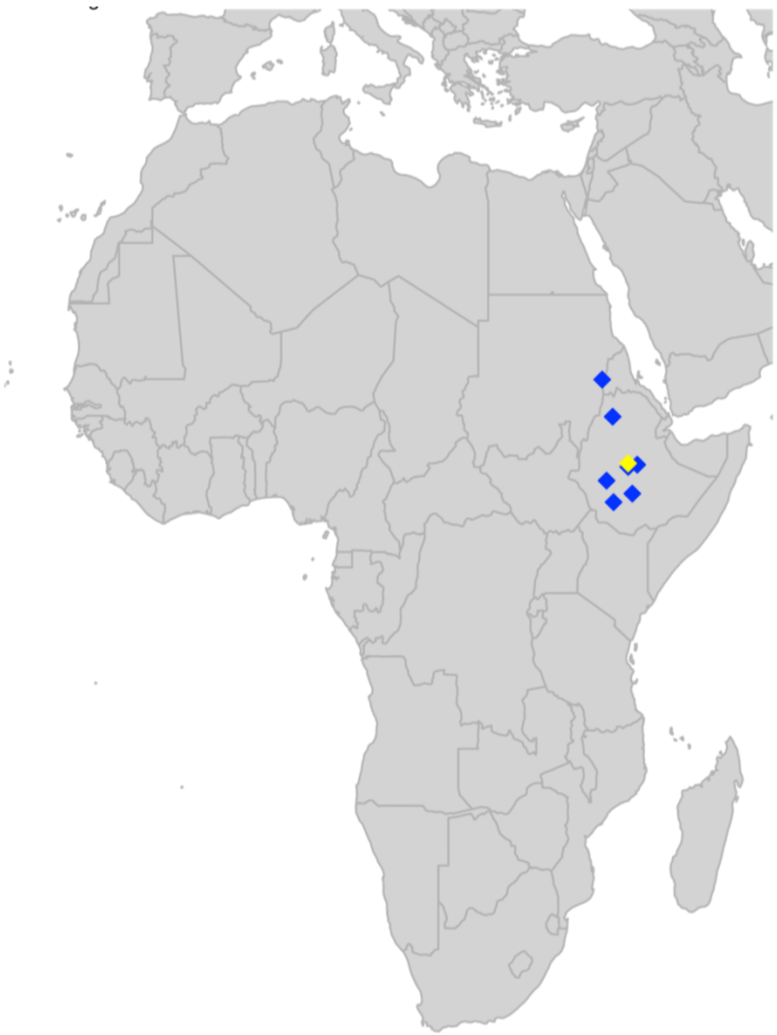

Figure S1. Study sites for efficacy studies – Africa Region

Blue – included; Yellow – targeted but not included.

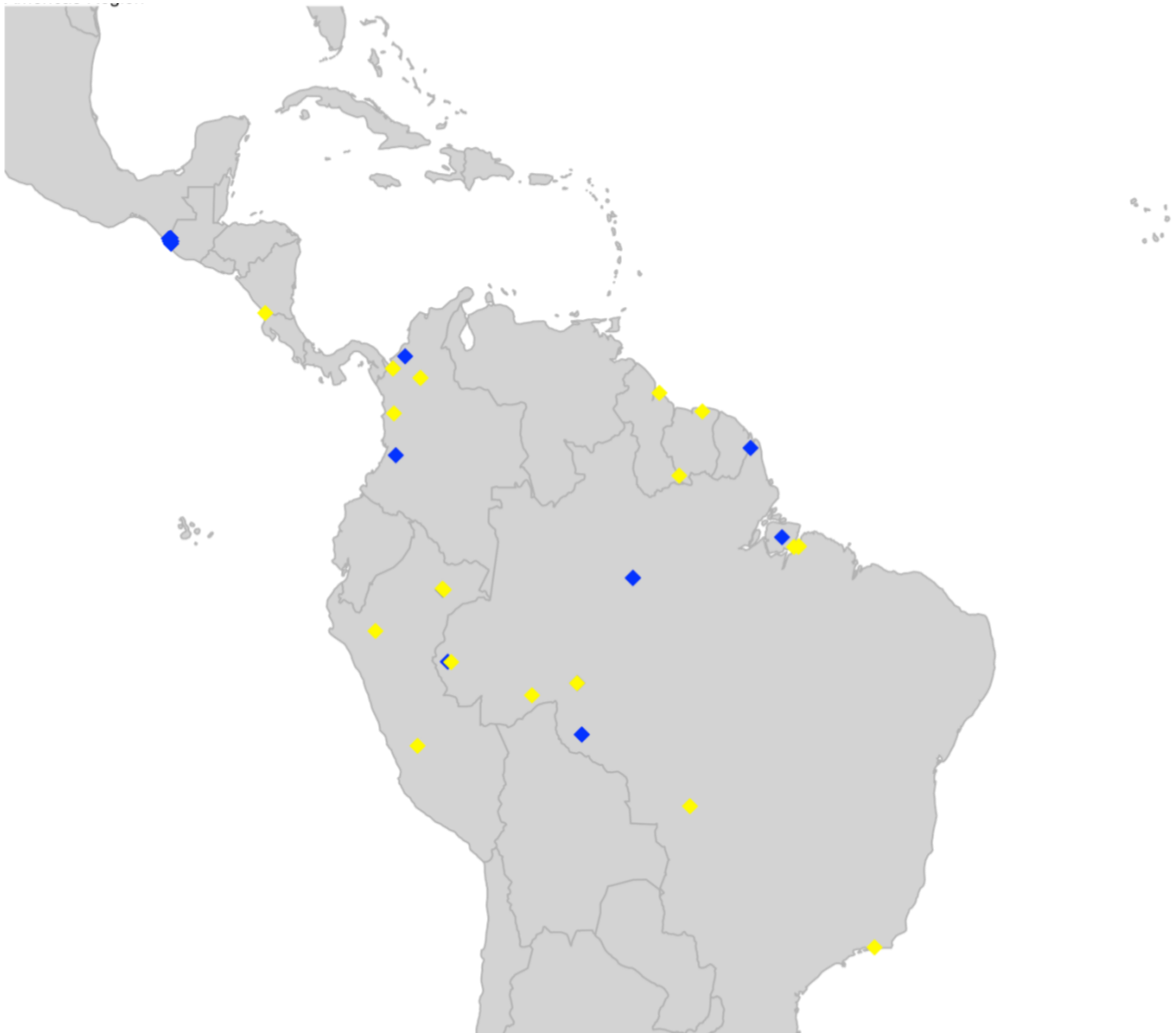

Figure S2. Study sites for efficacy studies – Americas Region

Blue – included; Yellow – targeted but not included.

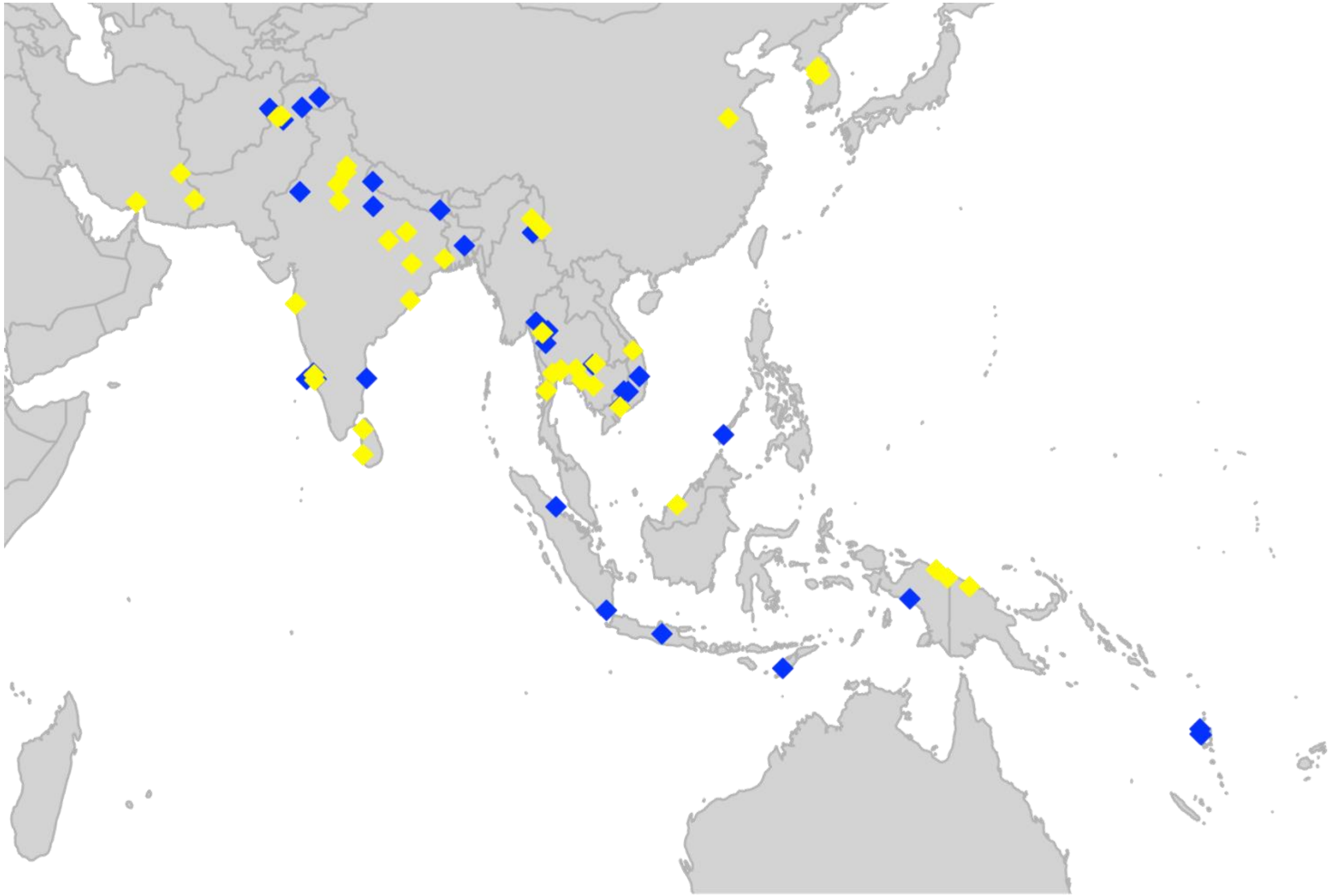

Figure S3. Study sites for efficacy studies – Asia Pacific Region

Blue – included; Yellow – targeted but not included.

Table S4. Comparison of baseline characteristics between included and targeted studies

Characteristic	Included studies (n=32†)	Targeted studies but not included (n=48 ◊)
**Region**		
Asia-Pacific, studies (%)	22 (59.5 %)	28 (58.3%)
Africa, studies (%)	3 (8.1%)	1 (2.1%)
The Americas, studies (%)	12 (32.4 %)	19 (39.6%)
**Year of enrolment**		
Pre-2010, studies (%)	2 (6.3 %)	23 (48.0%)
2010-2020, studies (%)	30 (94.8 %)	25 (52.0%)
**Age (years), median (IQR)**	19 (11-32)	27.1 (24.7-31.5) #
**Female, % of patients**	36.6%	30.4%*

IQR – inter-quartile range

◊ The studies with no information on adherence were not included in this column (n=3).

†Multinational studies were allocated to the multiple regions, resulting in 37 studies based on region; 5 studies are multinational.

#Median age was not available for 26 studies. *Percentage of female was not available for 8 studies.

Table S5. Distribution (number and percentage) of patients in adherence categories by study for (A) supervision, (B) total mg/kg dose administered

A)

Study	Adherence by supervision (%)		
	≤50 (N=2,790)	≥90 (N=4,127)	Total (N=6,917)
Chu-2018	0 (0.0%)	186 (100.0%)	186 (100.0%)
Nelwan-2015	0 (0.0%)	120 (100.0%)	120 (100.0%)
Leslie-2008	0 (0.0%)	55 (100.0%)	55 (100.0%)
Thanh-2015	0 (0.0%)	260 (100.0%)	260 (100.0%)
Hasugian-2007	340 (100.0%)	0 (0.0%)	340 (100.0%)
Abdallah-2012	38 (100.0%)	0 (0.0%)	38 (100.0%)
Chu-2019	0 (0.0%)	654 (100.0%)	654 (100.0%)
Karunajeewa-2016	0 (0.0%)	23 (100.0%)	23 (100.0%)
Saravu-2016	156 (100.0%)	0 (0.0%)	156 (100.0%)
Ley-2016	55 (100.0%)	0 (0.0%)	55 (100.0%)
Gonzalez-Ceron-2015	0 (0.0%)	88 (100.0%)	88 (100.0%)
Rishikesh-2015	124 (100.0%)	0 (0.0%)	124 (100.0%)
Lidia-2015	51 (100.0%)	0 (0.0%)	51 (100.0%)
Marques-2014	0 (0.0%)	135 (100.0%)	135 (100.0%)
Zuluaga-Idarraga-2016	0 (0.0%)	85 (100.0%)	85 (100.0%)
Gomes-2015	0 (0.0%)	94 (100.0%)	94 (100.0%)
Abreha-2017	194 (100.0%)	0 (0.0%)	194 (100.0%)
Pereira-2016	88 (100.0%)	0 (0.0%)	88 (100.0%)
Daher-2018	264 (100.0%)	0 (0.0%)	264 (100.0%)
Longley-2016	0 (0.0%)	56 (100.0%)	56 (100.0%)
Xu-2020	0 (0.0%)	281 (100.0%)	281 (100.0%)
Taylor-2019	0 (0.0%)	1,871 (100.0%)	1,871 (100.0%)
Ladeia-Andrade-2019	0 (0.0%)	101 (100.0%)	101 (100.0%)
Rijal-2019	105 (100.0%)	0 (0.0%)	105 (100.0%)
Yuan-2015	594 (100.0%)	0 (0.0%)	594 (100.0%)
Llanos-Cuentas-2019	85 (100.0%)	0 (0.0%)	85 (100.0%)
Saravu-2018	50 (100.0%)	0 (0.0%)	50 (100.0%)
Lacerda-2019	129 (100.0%)	0 (0.0%)	129 (100.0%)
Llanos-Cuentas-2013	50 (100.0%)	0 (0.0%)	50 (100.0%)
Brasil-2018	190 (100.0%)	0 (0.0%)	190 (100.0%)
Awab-2017	277 (98.2%)	5 (1.8%)	282 (100.0%)

de Sena-2019	0 (0.0%)	113 (100.0%)	113 (100.0%)

There were no patients with adherence for supervision between >50-<90% (%).

B)

Study	Adherence by total mg/kg dose administered (%)			
	≤50 (N=347)	>50-<90 (N=88)	≥90 (N=3,271)	Total (N=3,706)
Chu-2018	0 (0.0%)	0 (0.0%)	186 (100.0%)	186 (100.0%)
Nelwan-2015	1 (0.8%)	8 (6.7%)	111 (92.5%)	120 (100.0%)
Thanh-2015	256 (98.5%)	3 (1.2%)	1 (0.4%)	260 (100.0%)
Chu-2019	2 (0.3%)	17 (2.6%)	635 (97.1%)	654 (100.0%)
Karunajeewa-2016	0 (0.0%)	8 (34.8%)	15 (65.2%)	23 (100.0%)
Ley-2016	1 (1.8%)	6 (10.9%)	48 (87.3%)	55 (100.0%)
Gonzalez-Ceron-2015	2 (2.3%)	5 (5.7%)	81 (92.0%)	88 (100.0%)
Zuluaga-Idarraga-2016	0 (0.0%)	1 (1.2%)	84 (98.8%)	85 (100.0%)
Gomes-2015	0 (0.0%)	0 (0.0%)	94 (100.0%)	94 (100.0%)
Longley-2016	56 (100.0%)	0 (0.0%)	0 (0.0%)	56 (100.0%)
Taylor-2019	29 (1.5%)	28 (1.5%)	1,814 (97.0%)	1,871 (100.0%)
Ladeia-Andrade-2019	0 (0.0%)	6 (5.9%)	95 (94.1%)	101 (100.0%)
de Sena-2019	0 (0.0%)	6 (5.3%)	107 (94.7%)	113 (100.0%)

Table S6: Risk factors for *Plasmodium vivax* recurrence between days 7 and 90 in patients with information on supervision

	Number of patients#	Number of recurrences by day 90	Univariable analyses †		Multivariable analyses*	
			Unadjusted HR (95% CI)	p-value	Adjusted HR (95% CI)	p-value
Adherence by supervision †						
>=90%	4,042	167	Ref	..	Ref	..
<=50%	2,431	186	2.6 (2.1, 3.2)	<0.001	2.3 (1.8, 3.0)	<0.001
Age (years)	6,471	353	1.0 (0.9, 1.0)	0.467	1.0 (0.9, 1.0)	0.046
Age category (years)						
>=15	4,063	213	Ref	..	..	..
5-15	1,999	93	0.9 (0.7, 1.2)	0.400	..	..
<5	409	47	2.4 (1.7, 3.3)	<0.001	..	..
Weight (kg)	5,678	298	1.0 (1.0, 1.0)	0.542	..	..
Sex						
Male	4,093	223	Ref	..	Ref	..
Female	2,379	130	1.0 (0.8, 1.2)	0.901	1.0 (0.8, 1.3)	0.862
Relapse periodicity						
High periodicity	3,515	155	Ref	..	..	..
Low periodicity	2,958	198	1.6 (1.3, 1.9)	<0.0001	..	..
Geographical region						
Asia-Pacific	4,505	175	Ref	..	..	..
Americas	1,315	139	3.2 (2.5, 4.0)	<0.01	..	..
Africa	653	39	1.0 (0.8, 1.6)	0.617	..	..
Transmission intensity of study site						
High	2,187	184	Ref	..	..	..
Moderate	2,544	123	0.7 (0.6, 0.9)	0.011	..	..
Low	1,742	46	0.3 (0.2, 0.4)	<0.001	..	..
Blood-stage drug elimination half-life						
Slow, >7 days	6,216	323	Ref	..	..	..
Intermediate, 1-7 days	245	29	2.3 (1.5, 3.3)	<0.001	..	..
Rapid, <1 day	12	1	2.7 (0.4, 19.0)	0.329	..	..
Primaquine regimen duration						
14 days	3,506	178	Ref	..	..	..
<14 days	2,967	175	1.5 (1.2, 1.8)	0.005	..	..
Start day of primaquine						
Day 2/3	649	54	Ref	..	..	..
Day 0/1	5,824	299	0.5 (0.3, 0.6)	<0.001	..	..
Planned primaquine total dose (mg/kg)	6,468	353	0.9 (0.8, 0.9)	<0.001	0.92 (0.9, 1.0)	0.010
Haemoglobin (g/dL)	5,208	279	0.9 (0.8, 1.0)	0.001	..	..
Parasitaemia, parasites per µL every 10-fold increase	6,161	320	1.1 (0.9, 1.3)	0.180	1.1 (1.0, 1.4)	0.142
Malnutrition◊						
No	252	29	Ref	..	..	..
Yes	80	9	1.0 (0.5, 2.2)	0.909	..	..
Fever at baseline, temperature > 37.5°C						
No	2,669	155	Ref	..	..	..
Yes	2,676	113	0.9 (0.7, 1.0)	0.214	..	..

HR: hazard ratio, 95% CI: 95% confidence interval.

* The assumption of proportional hazards held for the model by visual assessment for the adherence by supervision.

# Patients with follow-up more than 7 days were considered in the Cox regression analysis.

To examine the robustness of the parameter estimates, a sensitivity analysis was carried out by removing one study site at a time, which showed that the overall coefficient of variation for reduced adherence by supervision (≤50 versus ≥90) estimates in the multivariable model was minimal (appendix table S8).

◊ Malnutrition status – calculated for children aged<5 years of age (malnutrition status was missing for 77 patients in this analysis; 76 in ≤50 and one in ≥90).

Data were missing for age (2 patients; one in ≤50 and one in ≥90), sex (one patient in ≥90), baseline parasitaemia (312 patients; 208 in ≤50 and 104 in ≥90), planned primaquine total dose (5 patients; 5 in ≥90), weight (795 patients; 733 in ≤50 and 62 in ≥90), baseline haemoglobin (1265 patients; 756 in ≤50 and 509 in ≥90), and fever (1128 patients; 599 in ≤50 and 529 in ≥90).

Table S7. Sensitivity analyses for effect of adherence by supervision on *Plasmodium vivax* recurrence between days 7 to 90 restricted to randomised studies or observational studies

	Randomised studies	Observational studies
Variable	Adjusted HR† (95% CI)	Adjusted HR† (95% CI)
Adherence by supervision (%)		
≥90	1	1
≤50	2.6 (1.9, 3.4)	1.6 (1.0, 2.6)

HR – hazard ratio.

Table S8. Sensitivity analysis for effect of adherence by supervision on *Plasmodium vivax* recurrence between days 7 to 90

Variable	Adjusted HR† (95% CI)	Range of HR	5th and 95th percentiles of HR	Coefficient of Variation (%) *
Adherence by supervision (%)				
≥90	1	1	1	..
≤50	2.3 (1.8, 3.0)	1.8-3.4	2.2-2.4	6.9

HR – hazard ratio; † - All study sites; Sensitivity analysis was generated by removing each study site one at a time (total of 70 sites). *The coefficient of variation calculated as standard deviation divided by the mean of the estimates.

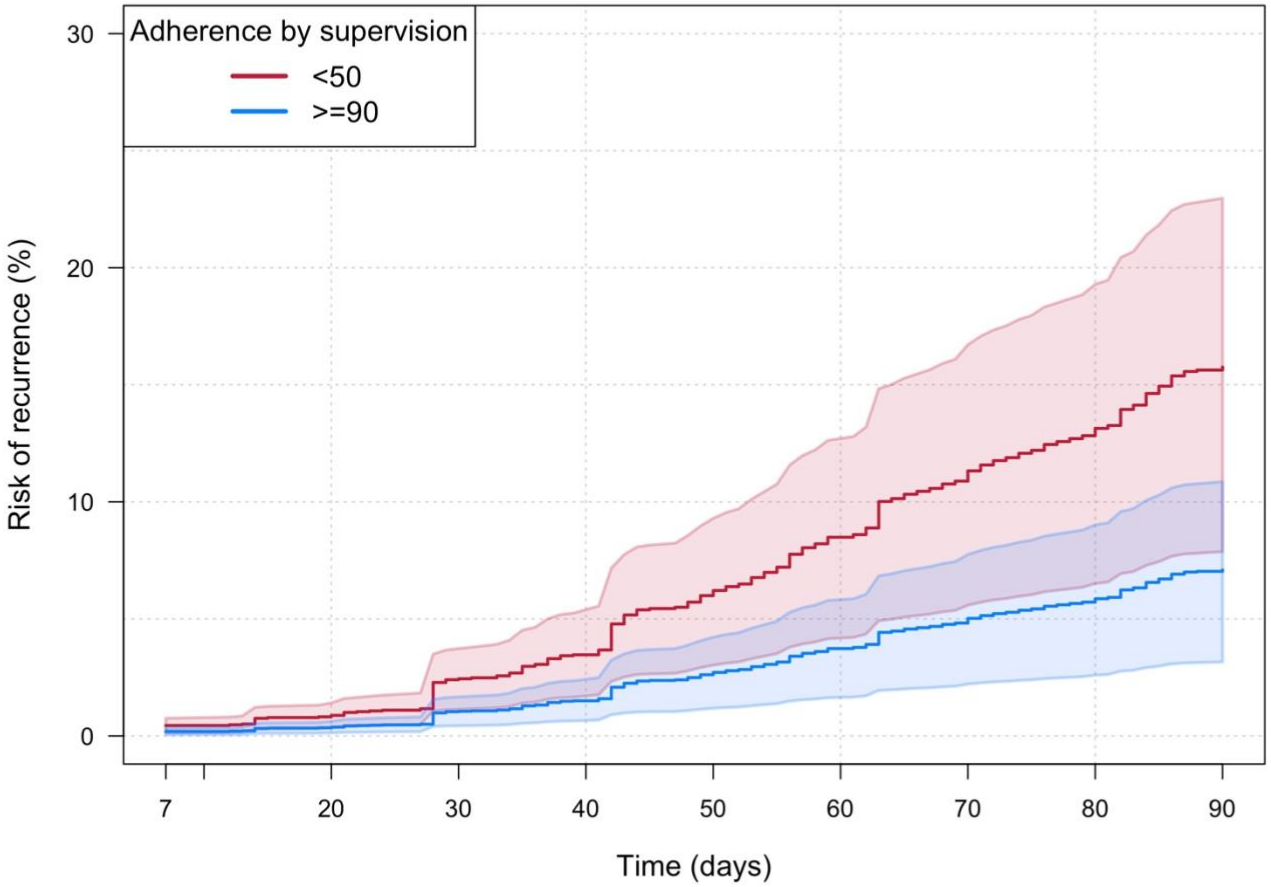

Figure S4: Adjusted risk of recurrence between days 7 and 90 in patients with information on supervision
Shaded regions represent the 95% CIs. Age, sex, planned primaquine total dose and baseline parasitaemia were set at baseline mean or prevalence. Assumes zero effect from study site.

Table S9: Demographic and baseline characteristics for adherence by total mg/kg dose administered				
	Adherence by total mg/kg dose administered (%)			
	≤50 (N=347)	>50-<90 (N=88)	≥90 (N=3,271)	Overall (N=3,706)
**Sex**				
Female	128 (36.9%)	28 (31.8%)	1,167 (35.7%)	1,323 (35.7%)
Male	219 (63.1%)	60 (68.2%)	2,103 (64.3%)	2,382 (64.3%)
**Age (years)**				
Median [IQR]	13.0 (7.0-24.0)	13.5 (9.0-25.0)	18.0 (11.0-29.0)	17.4 (11.0-29.0)
<5	44 (12.7%)	9 (10.2%)	204 (6.2%)	257 (6.9%)
5-15	150 (43.2%)	38 (43.2%)	1,048 (32.0%)	1,236 (33.4%)
>=15	153 (44.1%)	41 (46.6%)	2,019 (61.7%)	2,213 (59.7%)
**Relapse periodicity**				
Low periodicity	18 (5.2%)	32 (36.4%)	1,244 (38.0%)	1,294 (34.9%)
High periodicity	329 (94.8%)	56 (63.6%)	2,027 (62.0%)	2,412 (65.1%)
**Geographical region**				
Africa	7 (2.0%)	2 (2.3%)	456 (13.9%)	465 (12.5%)
Americas	2 (0.6%)	18 (20.5%)	461 (14.1%)	481 (13.0%)
Asia-Pacific	338 (97.4%)	68 (77.3%)	2,354 (72.0%)	2,760 (74.5%)
**Transmission intensity of study site**				
Low	273 (78.7%)	36 (40.9%)	1,272 (38.9%)	1,581 (42.7%)
Moderate	58 (16.7%)	24 (27.3%)	1,012 (30.9%)	1,094 (29.5%)
High	16 (4.6%)	28 (31.8%)	987 (30.2%)	1,031 (27.8%)
**Blood-stage drug elimination half-life**				
Intermediate, 1-7 days	0 (0.0%)	8 (9.1%)	23 (0.7%)	31 (0.8%)
Slow, >7 days	347 (100.0%)	80 (90.9%)	3,248 (99.3%)	3,675 (99.2%)
**Primaquine regimen duration**				
<14 days	265 (76.4%)	40 (45.5%)	1,524 (46.6%)	1,829 (49.4%)
14 days	82 (23.6%)	48 (54.5%)	1,747 (53.4%)	1,877 (50.6%)
**Start day of primaquine**				
Day 0/1	346 (99.7%)	80 (90.9%)	3,143 (96.1%)	3,569 (96.3%)
Day 2/3	1 (0.3%)	8 (9.1%)	128 (3.9%)	137 (3.7%)
**Planned primaquine total dose**				
Very low dose	0 (0.0%)	0 (0.0%)	208 (6.4%)	208 (5.6%)
Low dose	63 (18.2%)	38 (43.2%)	1,212 (37.1%)	1,313 (35.4%)
High dose	284 (81.8%)	50 (56.8%)	1,851 (56.6%)	2,185 (59.0%)
**Malnutrition**				
Yes	23 (52.3%)	5 (55.6%)	35 (17.2%)	63 (24.5%)
No	21 (47.7%)	4 (44.4%)	169 (82.8%)	194 (75.5%)
**Fever at baseline, temperature > 37.5°C**				
Yes	166 (57.2%)	35 (47.9%)	1,156 (40.3%)	1,357 (42.0%)
No	124 (42.8%)	38 (52.1%)	1,709 (59.7%)	1,871 (58.0%)
**Weight (kg)**	26.0 (15.0-46.0)	40.5 (22.8-56.6)	48.0 (29.2-58.0)	47.0 (27.0-57.0)
**Haemoglobin (g/dL)**	11.8 (10.5-13.3)	12.6 (11.4-13.7)	12.7 (11.6-14.0)	12.7 (11.5-13.9)
**Parasitaemia, parasites per µL**	3360.0 (1160.0-7299)	2435.7 (808.0-5180)	3466.7 (896.0-10000)	3400.0 (907.4-9774.1)

Data are presented as median (IQR) for continuous measures, and n (%) for categorical measures.

IQR – Interquartile range; Data were missing for the following variables: sex (1 patient in the ≥90% group); fever at baseline (406 patients in the ≥90 group, 15 patients in >50-<90% and 57 patients in ≤50%), baseline parasitaemia (49 patients in ≥90% group and 15 patient in the ≤50% group), baseline haemoglobin (173 patients in ≥90% group, 6 patients in >50-<90% group and 58 patient in the ≤50% group), weight (56 patient in the ≤50% group). Malnutrition status – calculated for children aged<5 years of age.

Table S10: Risk factors for *Plasmodium vivax* recurrence between days 7 and 90 in patients with information on total mg/kg dose administered

	Number of patients[#]	Number of recurrences by day 90	Univariable analyses †		Multivariable analyses*	
			Unadjusted HR (95% CI)	p-value	Adjusted HR (95% CI)	p-value
Adherence by total mg/kg dose administered						
Risk of recurrence between days 7 and 56						
>=90%	691	49	Ref	..	Ref	..
>50-<90%	38	2	1.2 (0.3, 4.9)	0.832	1.1 (0.3, 4.7)	0.884
<=50%	253	14	8.0 (1.5, 41.1)	0.013	10.1 (1.9, 52.4)	0.006
Risk of recurrence between days 56 and 90						
>=90	2,540	78	Ref	..	Ref	..
>50-<90%	43	8	6.2 (2.7, 13.9)	<0.001	6.0 (2.6, 13.9)	<0.001
<=50	58	2	5.2 (0.7, 40.3)	0.116	3.3 (0.3, 38.6)	0.336
Overall risk of recurrence between days 7 and 90						
>=90	3,231	127	Ref	..	Ref	..
>50-<90%	81	10	3.3 (1.6, 6.8)	0.001	3.2 (1.5, 6.7)	0.003
<=50	311	16	7.2 (1.8, 29.6)	0.006	7.6 (1.9, 30.0)	0.004
Age (years)	3,623	153	1.0 (0.9, 1.0)	0.016	1.0 (0.9, 1.00)	0.072
Age category (years)						
>=15	2,164	85	Ref	..	..	..
5-<15	1,209	51	1.1 (0.7, 1.5)	0.738	..	..
<5	250	17	1.6 (0.9, 2.7)	0.127	..	..
Weight (kg)	3,567	151	1.0 (0.9, 1.0)	0.136	..	..
Sex						
Male	2,319	102	Ref	..	Ref	..
Female	1,303	51	0.8 (0.6, 1.1)	0.171	0.8 (0.6, 1.1)	0.161
Relapse periodicity						
High periodicity	2,378	80	Ref	..	..	..
Low periodicity	1,245	73	0.9 (0.3, 3.5)	0.908	..	..
Geographical region						
Asia-Pacific	2,704	97	Ref	..	..	..
Americas	469	35	1.6 (0.4, 6.6)	0.515	..	..
Africa	450	21	0.2 (0.0, 2.0)	0.171	..	..
Transmission intensity of study site						
High	987	68	Ref	..	..	..
Moderate	1,091	48	0.2 (0.0, 0.9)	0.042	..	..
Low	1,545	37	0.4 (0.1, 1.5)	0.159	..	..
Blood-stage drug elimination half-life						
Slow, >7 days	3,592	144	Ref	..	..	..
Intermediate, 1-7 days	31	9	1.7 (1.9-29.7)	0.424	..	..
Primaquine regimen duration						
14 days	1,848	71	Ref	..	..	..
<14 days	1,775	82	1.5 (1.0, 2.3)	0.047	..	..
Start day of primaquine						
Day 2/3	134	1	Ref	..	..	..
Day 0/1	3,489	152	3.2 (0.4, 23.9)	0.264	..	..

**Haemoglobin (g/dL)**	3,391	139	0.9 (0.9, 1.0)	0.297	..	..
**Parasitaemia, parasites per µL every 10-fold increase**	3,574	148	1.2 (0.9, 1.5)	0.248	1.1 (0.9, 1.4)	0.429
**Malnutrition**						
No	189	10	Ref	..	..	..
Yes	61	7	2.4 (0.9, 6.6)	0.089	..	..
**Fever at baseline, temperature > 37.5°C**						
No	1,833	79	Ref	..	..	..
Yes	1,317	54	1.1 (0.8, 1.6)	0.527	..	..

HR: hazard ratio, 95% CI: 95% confidence interval.

# Patients with follow-up more than 7 days were considered in the Cox regression analysis.

† Data were missing for sex (one patient; one in the ≥90), baseline parasitaemia (49 patients; 14 in ≤50 and 35 in the ≥90), fever (473 patients; 57 in ≤50, 15 in >50-<90 and 401 in the ≥90), weight (56 patients; 56 in ≤50), and baseline haemoglobin (232 patients; 58 in ≤50, 6 in >50-<90 and 168 in the ≥90).

The adjusted HRs for age, sex, and parasitaemia are presented for the total mg/kg dose model assumes constant HR over follow-up period.

* The effect of study sites was not assessed due to collinearity with adherence. The proportional hazards assumption did not hold on visual inspection of the exposure of interest, adherence by total mg/kg dose. To examine the robustness of the parameter estimates, a sensitivity analysis was carried out by removing one study site at a time, which showed that the overall coefficient of variation for levels of adherence estimates in the multivariable model were small (Appendix Table S11).

Malnutrition status – calculated for children aged<5 years of age.

Table S11. Sensitivity analysis for effect of adherence by total dose (mg/kg) administered on *P. vivax* recurrence between days 7 to 90

Variable	Adjusted HR† (95% CI)	Range of HR	5th and 95th percentiles of HR	Coefficient of Variation (%)*
Adherence by total dose administered (%)				
≥90	1	1	1	..
>50-<90	3.2 (1.5, 6.7)	2.5-5.1	2.6-3.9	14.0
≤50	7.6 (1.9, 30.0)	2.9-10.7†	4.6-10.0	18.7

HR – hazard ratio; † - All study sites; Sensitivity analysis was generated by removing each study site one at a time (total of 27 sites).
*The coefficient of variation calculated as standard deviation divided by the mean of the estimates.
†Thanh-2015 and Taylor-2019 are the two studies that have the greatest influence on changing the magnitude of the HR.

Table S12: Demographic and baseline characteristics for adherence by actual dosing			
	Adherence by actual dosing (%)		
	≥90 (N=2,824)	<90 (N=86)	Overall (N=2,910)
**Sex**			
Female	1,009 (35.7%)	23 (26.7%)	1,032 (35.5%)
Male	1,815 (64.3%)	63 (73.3%)	1,878 (64.5%)
**Age (years)**			
Median [IQR]	17.9 (11.0-28.0)	19.7 (12.0-29.0)	18.0 (11.0-28.1)
<5	168 (5.9%)	6 (7.0%)	174 (6.0%)
5-<15	930 (32.9%)	23 (26.7%)	953 (32.7%)
>=15	1,726 (61.1%)	57 (66.3%)	1,783 (61.3%)
**Relapse periodicity**			
Low periodicity	778 (27.5%)	35 (40.7%)	813 (27.9%)
High periodicity	2,046 (72.5%)	51 (59.3%)	2,097 (72.1%)
**Geographical region**			
Africa	454 (16.1%)	11 (12.8%)	465 (16.0%)
Asia-Pacific	2,370 (83.9%)	75 (87.2%)	2,445 (84.0%)
**Transmission intensity of study site**			
Low	1,151 (40.8%)	27 (31.4%)	1,178 (40.5%)
Moderate	873 (30.9%)	23 (26.7%)	896 (30.8%)
High	800 (28.3%)	36 (41.9%)	836 (28.7%)
**Blood-stage drug elimination half-life**			
Intermediate, 1-7 days	30 (1.1%)	1 (1.2%)	31 (1.1%)
Slow, >7 days	2,794 (98.9%)	85 (98.8%)	2,879 (98.9%)
**Primaquine regimen duration**			
<14 days	1,220 (43.2%)	41 (47.7%)	1,261 (43.3%)
14 days	1,604 (56.8%)	45 (52.3%)	1,649 (56.7%)
**Start day of primaquine**			
Day 0/1	2,744 (97.2%)	84 (97.7%)	2,828 (97.2%)
Day 2/3	80 (2.8%)	2 (2.3%)	82 (2.8%)
**Planned primaquine total dose**			
Very low dose	191 (6.8%)	5 (5.8%)	196 (6.7%)
Low dose	790 (28.0%)	31 (36.0%)	821 (28.2%)
High dose	1,843 (65.3%)	50 (58.1%)	1,893 (65.1%)
**Malnutrition**			
Yes	35 (20.8%)	3 (50.0%)	38 (21.8%)
No	133 (79.2%)	3 (50.0%)	136 (78.2%)
**Fever at baseline, temperature > 37.5°C**			
Yes	1,032 (39.1%)	38 (45.8%)	1,070 (39.3%)
No	1,609 (60.9%)	45 (54.2%)	1,654 (60.7%)
**Weight (kg)**	47.0 (28.0-56.6)	51.4 (29.5-58.5)	47.0 (28.0-56.7)
**Haemoglobin (g/dL)**	12.8 (11.6-14.0)	13.1 (11.6-14.6)	12.8 (11.6-14.0)
**Parasitaemia, parasites per µL**	3814.8 (973.6-11304.0)	4059.3 (1408.0-7129.6)	3837.0 (992.0-11244.4)

Data are presented as median (IQR) for continuous measures, and n (%) for categorical measures.

IQR – Interquartile range; Data were missing for the following variables: baseline parasitaemia (56 patients in ≥90% group and one patient in the <90% group), baseline haemoglobin (61 patients in ≥90% group and 2 patient in the <90% group), weight (54 patients in ≥90% group and 2 patient in the <90% group), fever at baseline (183 patients in the ≥90% group and 3 patients in <90%), vomiting (992 patients in the ≥90% group and 24 patients in <90%). Malnutrition status – calculated for children aged<5 years of.

References S1. Studies not included in analysis

60. Dharmawardena P, Rodrigo C, Mendis K, de AWGW, Premaratne R, Ringwald P, et al. Response of imported malaria patients to antimalarial medicines in Sri Lanka following malaria elimination. PLoS One. 2017/11/29. 2017;12(11): e0188613. https://doi.org/10.1371/journal.pone.0188613.
61. Carmona-Fonseca J, Maestre A. Prevention of Plasmodium vivax malaria recurrence: efficacy of the standard total dose of primaquine administered over 3 days. Acta Trop. 2009/08/06. 2009;112(2): 188–192. https://doi.org/10.1016/j.actatropica.2009.07.024.
62. Carmona-Fonseca J. Vivax malaria in children: Recurrences with standard total dose of primaquine administered in 3 vs. 7 days. Iatreia. 2010;23(1): 10–20.
63. Valecha N, Savargaonkar D, Srivastava B, Rao BH, Tripathi SK, Gogtay N, et al. Comparison of the safety and efficacy of fixed-dose combination of arterolane maleate and piperaquine phosphate with chloroquine in acute, uncomplicated Plasmodium vivax malaria: a phase III, multicentric, open-label study. Malar J. 2016/01/29. 2016;15: 42. https://doi.org/10.1186/s12936-016-1084-1.
64. da Silva Rdo S, Pinto AY, Calvosa VS, de Souza JM. [Short course schemes for vivax malaria treatment]. Rev Soc Bras Med Trop. 2003/06/14. 2003;36(2): 235–239. https://www.ncbi.nlm.nih.gov/pubmed/12806460
65. Eibach D, Ceron N, Krishnalall K, Carter K, Bonnot G, Bienvenu AL, et al. Therapeutic efficacy of artemether-lumefantrine for Plasmodium vivax infections in a prospective study in Guyana. Malar J. 2012/10/23. 2012;11: 347. https://doi.org/10.1186/1475-2875-11-347.
66. Bergonzoli G, Rivers Cuadra JC. [Therapeutic efficacy of different antimalarial regimens in the Costa Rica-Nicaragua border region]. Rev Panam Salud Publica. 2000/08/19. 2000;7(6): 366–370. https://www.ncbi.nlm.nih.gov/pubmed/10949896
67. Lee SW, Lee M, Lee DD, Kim C, Kim YJ, Kim JY, et al. Biological resistance of hydroxychloroquine for Plasmodium vivax malaria in the Republic of Korea. Am J Trop Med Hyg. 2009/10/10. 2009;81(4): 600–604. https://doi.org/10.4269/ajtmh.2009.09-0102.
68. Krudsood S, Wilairatana P, Tangpukdee N, Chalermrut K, Srivilairit S, Thanachartwet V, et al. Safety and tolerability of elubaquine (bulaquine, CDRI 80/53) for treatment of Plasmidium vivax malaria in Thailand. Korean J Parasitol. 2006/09/14. 2006;44(3): 221–228. https://www.ncbi.nlm.nih.gov/pubmed/16969059
69. Mohapatra MK, Padhiary KN, Mishra DP, Sethy G. Atypical manifestations of Plasmodium vivax malaria. Indian J Malariol. 2003/12/23. 2002;39(1–2): 18–25. https://www.ncbi.nlm.nih.gov/pubmed/14686106
70. Orjuela-Sanchez P, da Silva NS, da Silva-Nunes M, Ferreira MU. Recurrent parasitemias and population dynamics of Plasmodium vivax polymorphisms in rural Amazonia. Am J Trop Med Hyg. 2009;81(6): 961–968. https://doi.org/10.4269/ajtmh.2009.09-0337.
71. Alvarez G, Pineros JG, Tobon A, Rios A, Maestre A, Blair S, et al. Efficacy of three chloroquine-primaquine regimens for treatment of Plasmodium vivax malaria in Colombia. Am J Trop Med Hyg. 2006/10/14. 2006;75(4): 605–609. https://www.ncbi.nlm.nih.gov/pubmed/17038680
72. Leslie T, Rab MA, Ahmadzai H, Durrani N, Fayaz M, Kolaczinski J, et al. Compliance with 14-day primaquine therapy for radical cure of vivax malaria--a randomized placebo-controlled trial comparing unsupervised with supervised treatment. Trans R Soc Trop Med Hyg. 2004/03/18. 2004;98(3): 168–173. https://www.ncbi.nlm.nih.gov/pubmed/15024927
73. Rowland M, Durrani N. Randomized controlled trials of 5- and 14-days primaquine therapy against relapses of vivax malaria in an Afghan refugee settlement in Pakistan. Trans R Soc Trop Med Hyg. 2000/03/16. 1999;93(6): 641–643. https://www.ncbi.nlm.nih.gov/pubmed/10717755
74. mac Donald-Ottevanger MS, Adhin MR, Jitan JK, Bretas G, Vreden SG. Primaquine double dose for 7 days is inferior to single-dose treatment for 14 days in preventing Plasmodium vivax recurrent episodes in Suriname. Infect Drug Resist. 2018/01/11. 2018;11: 3–8. https://doi.org/10.2147/IDR.S135897.
75. Mesa-Echeverry E, Niebles-Bolívar M, Tobón-Castaño A. Chloroquine-Primaquine Therapeutic Efficacy, Safety, and Plasma Levels in Patients with Uncomplicated Plasmodium vivax Malaria in a Colombian Pacific Region. The American journal of tropical medicine and hygiene. 2019;100(1): 72–77. https://doi.org/10.4269/AJTMH.18-0655.
76. Han JH, Cho JS, Ong JJY, Park JH, Nyunt MH, Sutanto E, et al. Genetic diversity and neutral selection in Plasmodium vivax erythrocyte binding protein correlates with patient antigenicity. PLOS Neglected Tropical Diseases. 2020;14(7): e0008202. https://doi.org/10.1371/JOURNAL.PNTD.0008202.
77. Villalobos-Salcedo JM, Tada MS, Kimura E, Menezes MJ, Pereira da Silva LH. In-vivo sensitivity of Plasmodium vivax isolates from Rond nia (western Amazon region, Brazil) to regimens including chloroquine and primaquine. Ann Trop Med Parasitol. 2001/02/24. 2000;94(8): 749–758. https://www.ncbi.nlm.nih.gov/pubmed/11214093
78. Abdon NP, Pinto AY, das Silva Rdo S, de Souza JM. [Assessment of the response to reduced treatment schemes for vivax malaria]. Rev Soc Bras Med Trop. 2001/09/20. 2001;34(4): 343–348. https://www.ncbi.nlm.nih.gov/pubmed/11562727

79. Dua VK, Sharma VP. Plasmodium vivax relapses after 5 days of primaquine treatment, in some industrial complexes of India. Ann Trop Med Parasitol. 2002/01/11. 2001;95(7): 655–659. https://doi.org/10.1080/00034980120103225.
80. Duarte EC, Pang LW, Ribeiro LC, Fontes CJ. Association of subtherapeutic dosages of a standard drug regimen with failures in preventing relapses of vivax malaria. Am J Trop Med Hyg. 2001;65(5): 471–476. http://www.ncbi.nlm.nih.gov/pubmed/11716100
81. Yadav RS, Ghosh SK. Radical curative efficacy of five-day regimen of primaquine for treatment of Plasmodium vivax malaria in India. J Parasitol. 2002/11/19. 2002;88(5): 1042–1044. https://doi.org/10.1645/0022-3395(2002)088[1042:RCEOFD]2.0.CO;2.
82. Machado RL, de Figuereido Filho AF, Calvosa VS, Figueredo MC, Nascimento JM, Povoa MM. Correlation between Plasmodium vivax variants in Belem, Para State, Brazil and symptoms and clearance of parasitaemia. Braz J Infect Dis. 2003/09/23. 2003;7(3): 175–177. https://www.ncbi.nlm.nih.gov/pubmed/14499040
83. Pinto AY, Azevedo CH, da Silva JB, de Souza JM. Assessment of chloroquine single dose treatment of malaria due to Plasmodium vivax in Brazilian Amazon. Rev Inst Med Trop Sao Paulo. 2004/02/06. 2003;45(6): 327–331. https://www.ncbi.nlm.nih.gov/pubmed/14762633
84. Hapuarachchi HA, Dayanath MY, Abeysundara S, Bandara KB, Abeyewickreme W, de Silva NR. Chloroquine resistant falciparum malaria among security forces personnel in the Northern Province of Sri Lanka. Ceylon Med J. 2004/09/01. 2004;49(2): 47–51. https://www.ncbi.nlm.nih.gov/pubmed/15334798
85. Maguire JD, Krisin, Marwoto H, Richie TL, Fryauff DJ, Baird JK. Mefloquine is highly efficacious against chloroquine-resistant Plasmodium vivax malaria and Plasmodium falciparum malaria in Papua, Indonesia. Clin Infect Dis. 2006/04/01. 2006;42(8): 1067–1072. https://doi.org/10.1086/501357.
86. Takeuchi R, Lawpoolsri S, Imwong M, Kobayashi J, Kaewkungwal J, Pukrittayakamee S, et al. Directly-observed therapy (DOT) for the radical 14-day primaquine treatment of Plasmodium vivax malaria on the Thai-Myanmar border. Malar J. 2010/11/03. 2010;9: 308. https://doi.org/10.1186/1475-2875-9-308.
87. Yeshiwondim AK, Tekle AH, Dengela DO, Yohannes AM, Teklehaimanot A. Therapeutic efficacy of chloroquine and chloroquine plus primaquine for the treatment of Plasmodium vivax in Ethiopia. Acta Trop. 2009/10/20. 2010;113(2): 105–113. https://doi.org/10.1016/j.actatropica.2009.10.001.
88. Muhamad P, Ruengweerayut R, Chacharoenkul W, Rungsihirunrat K, Na-Bangchang K. Monitoring of clinical efficacy and in vitro sensitivity of Plasmodium vivax to chloroquine in area along Thai Myanmar border during 2009-2010. Malar J. 2011/02/18. 2011;10: 44. https://doi.org/10.1186/1475-2875-10-44.
89. Graf PC, Durand S, Alvarez Antonio C, Montalvan C, Galves Montoya M, Green MD, et al. Failure of Supervised Chloroquine and Primaquine Regimen for the Treatment of Plasmodium vivax in the Peruvian Amazon. Malar Res Treat. 2012/06/16. 2012;2012: 936067. https://doi.org/10.1155/2012/936067.
90. Pedro RS, Guaraldo L, Campos DP, Costa AP, Daniel-Ribeiro CT, Brasil P. Plasmodium vivax malaria relapses at a travel medicine centre in Rio de Janeiro, a non-endemic area in Brazil. Malar J. 2012/07/31. 2012;11: 245. https://doi.org/10.1186/1475-2875-11-245.
91. Zhu G, Lu F, Cao J, Zhou H, Liu Y, Han ET, et al. Blood stage of Plasmodium vivax in central China is still susceptible to chloroquine plus primaquine combination therapy. Am J Trop Med Hyg. 2013/05/15. 2013;89(1): 184–187. https://doi.org/10.4269/ajtmh.12-0683.
92. Delgado-Ratto C, Soto-Calle VE, van den Eede P, Gamboa D, Rosas A, Abatih EN, et al. Population structure and spatio-temporal transmission dynamics of Plasmodium vivax after radical cure treatment in a rural village of the Peruvian Amazon. Malar J. 2014/01/08. 2014;13: 8. https://doi.org/10.1186/1475-2875-13-8.
93. Cheoymang A, Ruenweerayut R, Muhamad P, Rungsihirunrat K, Na-Bangchang K. Patients' adherence and clinical effectiveness of a 14-day course of primaquine when given with a 3-day chloroquine in patients with Plasmodium vivax at the Thai-Myanmar border. Acta Trop. 2015/08/19. 2015;152: 151–156. https://doi.org/10.1016/j.actatropica.2015.08.008.
94. Pukrittayakamee S, Chantra A, Simpson JA, Vanijanonta S, Clemens R, Looareesuwan S, et al. Therapeutic responses to different antimalarial drugs in vivax malaria. Antimicrob Agents Chemother. 2000/05/19. 2000;44(6): 1680–1685. https://www.ncbi.nlm.nih.gov/pubmed/10817728
95. Buchachart K, Krudsood S, Singhasivanon P, Treeprasertsuk S, Phophak N, Srivilairit S, et al. Effect of primaquine standard dose (15 mg/day for 14 days) in the treatment of vivax malaria patients in Thailand. Southeast Asian J Trop Med Public Health. 2002/06/04. 2001;32(4): 720–726. https://www.ncbi.nlm.nih.gov/pubmed/12041544
96. Tasanor O, Ruengweerayut R, Sirichaisinthop J, Congpuong K, Wernsdorfer WH, Na-Bangchang K. Clinical-parasitological response and in-vitro sensitivity of Plasmodium vivax to chloroquine and quinine on the western border of Thailand. Trans R Soc Trop Med Hyg. 2006/02/25. 2006;100(5): 410–418. https://doi.org/10.1016/j.trstmh.2005.04.024.

97. Krudsood S, Tangpukdee N, Muangnoicharoen S, Thanachartwet V, Luplertlop N, Srivilairit S, et al. Clinical efficacy of chloroquine versus artemether-lumefantrine for Plasmodium vivax treatment in Thailand. Korean J Parasitol. 2007/06/16. 2007;45(2): 111–114. https://www.ncbi.nlm.nih.gov/pubmed/17570973
98. Ganguly S, Saha P, Guha SK, Das S, Bera DK, Biswas A, et al. In vivo therapeutic efficacy of chloroquine alone or in combination with primaquine against vivax malaria in Kolkata, West Bengal, India, and polymorphism in pvmdr1 and pvcrt-o genes. Antimicrob Agents Chemother. 2012/12/25. 2013;57(3): 1246–1251. https://doi.org/10.1128/AAC.02050-12.
99. Liu H, Yang HL, Xu JW, Wang JZ, Nie RH, Li CF. Artemisinin-naphthoquine combination versus chloroquine-primaquine to treat vivax malaria: an open-label randomized and non-inferiority trial in Yunnan Province, China. Malar J. 2013/11/13. 2013;12: 409. https://doi.org/10.1186/1475-2875-12-409.
100. Macareo L, Lwin KM, Cheah PY, Yuentrakul P, Miller RS, Nosten F. Triangular test design to evaluate tinidazole in the prevention of Plasmodium vivax relapse. Malar J. 2013/05/31. 2013;12: 173. https://doi.org/10.1186/1475-2875-12-173.
101. Pareek A, Chandurkar N, Gogtay N, Deshpande A, Kakrani A, Kaneria M, et al. Sustained Release Formulation of Primaquine for Prevention of Relapse of Plasmodium vivax Malaria: A Randomized, Double-Blind, Comparative, Multicentric Study. Malar Res Treat. 2015/09/15. 2015;2015: 579864. https://doi.org/10.1155/2015/579864.
102. Daneshvar C, Davis TM, Cox-Singh J, Rafa'ee MZ, Zakaria SK, Divis PC, et al. Clinical and parasitological response to oral chloroquine and primaquine in uncomplicated human Plasmodium knowlesi infections. Malar J. 2010/08/21. 2010;9: 238. https://doi.org/10.1186/1475-2875-9-238.
103. van den Eede P, Soto-Calle VE, Delgado C, Gamboa D, Grande T, Rodriguez H, et al. Plasmodium vivax sub-patent infections after radical treatment are common in Peruvian patients: results of a 1-year prospective cohort study. PLoS One. 2011/02/08. 2011;6(1): e16257. https://doi.org/10.1371/journal.pone.0016257.
104. Negreiros S, Farias S, Viana GM, Okoth SA, Chenet SM, de Souza TM, et al. Efficacy of Chloroquine and Primaquine for the Treatment of Uncomplicated Plasmodium vivax Malaria in Cruzeiro do Sul, Brazil. Am J Trop Med Hyg. 2016/11/04. 2016;95(5): 1061–1068. https://doi.org/10.4269/ajtmh.16-0075.
105. Fukuda MM, Krudsood S, Mohamed K, Green JA, Warrasak S, Noedl H, et al. A randomized, double-blind, active-control trial to evaluate the efficacy and safety of a three day course of tafenoquine monotherapy for the treatment of Plasmodium vivax malaria. PLoS One. 2017/11/10. 2017;12(11): e0187376. https://doi.org/10.1371/journal.pone.0187376.
106. Pham TV, van Nguyen H, Aguirre AR, van Nguyen V, Cleves MA, Nguyen XX, et al. Plasmodium vivax morbidity after radical cure: A cohort study in Central Vietnam. PLOS Medicine. 2019;16(5): e1002784. https://doi.org/10.1371/JOURNAL.PMED.1002784.
107. Maneeboonyang W, Lawpoolsri S, Puangsa-Art S, Yimsamran S, Thanyavanich N, Wuthisen P, et al. Directly observed therapy with primaquine to reduce the recurrence rate of plasmodium vivax infection along the Thai-Myanmar border. Southeast Asian J Trop Med Public Health. 2011;42(1): 9–18. https://www.ncbi.nlm.nih.gov/pubmed/21323159
108. Pasaribu AP, Chokejindachai W, Sirivichayakul C, Tanomsing N, Chavez I, Tjitra E, et al. A randomized comparison of dihydroartemisinin-piperaquine and artesunate-amodiaquine combined with primaquine for radical treatment of vivax malaria in Sumatera, Indonesia. J Infect Dis. 2013/08/09. 2013;208(11): 1906–1913. https://doi.org/10.1093/infdis/jit407.
109. Barber BE, William T, Grigg MJ, Menon J, Auburn S, Marfurt J, et al. A prospective comparative study of knowlesi, falciparum, and vivax malaria in Sabah, Malaysia: high proportion with severe disease from Plasmodium knowlesi and Plasmodium vivax but no mortality with early referral and artesunate therapy. Clin Infect Dis. 2013;56(3): 383–397. https://doi.org/10.1093/cid/cis902.
110. Grigg MJ, William T, Menon J, Barber BE, Wilkes CS, Rajahram GS, et al. Efficacy of Artesunate-mefloquine for Chloroquine-resistant Plasmodium vivax Malaria in Malaysia: An Open-label, Randomized, Controlled Trial. Clin Infect Dis. 2016;62(11): 1403–1411. https://doi.org/10.1093/cid/ciw121.
